# Continuous-Wave Electron Paramagnetic Resonance Spectroscopy in Pharmaceutical Research: Current Applications and Emerging Opportunities

**DOI:** 10.3390/ph19091424

**Published:** 2026-09-09

**Authors:** Erim Bešić, Davor Šakić

**Affiliations:** 1 Department of Biophysics, Faculty of Pharmacy and Biochemistry, University of Zagreb, 10000 Zagreb, Croatia; 2 Department of Analytical Chemistry, Faculty of Pharmacy and Biochemistry, University of Zagreb, 10000 Zagreb, Croatia; davor.sakic@pharma.unizg.hr

**Keywords:** continuous-wave EPR, free radicals, spin trapping, spin labeling, spin probes, antioxidant activity, drug-induced toxicity, drug delivery, metallodrugs, EPR oximetry

## Abstract

**Background/Objectives**: Electron paramagnetic resonance (EPR) spectroscopy is a direct and highly specific method for detecting paramagnetic species, including free radicals, transition-metal centers, spin labels and spin probes. In pharmaceutical research, it is particularly valuable in systems involving radical chemistry, oxidative degradation, drug–membrane interactions, drug delivery carriers, metallodrugs and oxygen-sensitive microenvironments. This review aims to summarize current applications of and emerging opportunities for EPR spectroscopy in pharmaceutical research, with primary emphasis on continuous-wave (CW) EPR, the mode most widely used for routine pharmaceutical measurements. **Methods**: This review is organized around the main CW-EPR approaches relevant to pharmaceutical research, including direct detection of paramagnetic species, spin trapping, spin labeling and spin-probe analysis. The discussion emphasizes how spectral parameters such as the g-value, hyperfine splitting, signal intensity and linewidth can provide structural, kinetic and microenvironmental information. **Results**: CW-EPR supports the study of radical-mediated drug activity and toxicity, antioxidant properties, formulation stability, membrane interactions, carrier structure, metallodrug behavior and oxygenation. Its main strength is the ability to connect molecular-level radicals and paramagnetic processes with broader pharmaceutical questions related to drug efficacy, safety, stability and delivery. **Conclusions**: CW-EPR is a specialized but versatile analytical tool at the interface of radical chemistry, pharmaceutical technology and biomedical research. Recent developments in compact instrumentation, selective probes and spectral simulation may further expand its use in pharmaceutical development, stability assessment and drug delivery research.

## 1. Introduction

Interest in free radicals in biomedical sciences has increased markedly because these species have a dual role in living systems. Reactive oxygen and nitrogen species participate in cellular signaling, regulation of inflammation and host defense, but excessive radical formation overwhelms antioxidant protection and leads to oxidative stress. Such imbalance can damage lipids, proteins, nucleic acids and biological membranes and is associated with inflammation, ischemia–reperfusion injury, aging, neurodegenerative diseases, atherosclerosis, diabetes and cancer [1,2,3]. This dual biological role explains why methods capable of detecting radicals directly are of particular importance for pharmaceutical research.

Many questions encountered during drug discovery, formulation development and quality control cannot be fully resolved by conventional chromatographic, spectrophotometric or electrochemical procedures alone. In a number of cases, the decisive species are short-lived radicals, redox-active intermediates, oxidative degradation products or local changes within membrane and polymeric microenvironments. Electron paramagnetic resonance (EPR) spectroscopy is especially valuable in such circumstances because it directly detects paramagnetic species, that is, molecules, ions or complexes containing an unpaired electron. This capability is highly relevant in studies of antioxidant activity, mechanisms of anticancer and antimalarial drugs, photostability, sterilization-induced radical formation and oxidative changes during storage of pharmaceutical preparations [4,5,6]. In pharmaceutical systems, free radical formation may also arise from autoxidation, metal-catalyzed oxidation, photodegradation and mechanically induced stress [7]. This further supports the need for methods that can detect radical species directly.

In pharmaceutical analysis, EPR spectroscopy can be applied at several stages of drug development. In early screening, it helps identify compounds that scavenge free radicals or generate radical intermediates. During formulation development, it may be used to evaluate stability, examine drug–membrane interactions or follow drug release from nano- and microcarriers. In quality control, it can detect paramagnetic impurities, degradation products and changes caused by irradiation, thermal treatment or sterilization. In biomedical models, EPR-based approaches can monitor tissue oxygenation, pH-sensitive probes and the reducing capacity of the tumor microenvironment [4,8]. Recent studies further support the use of EPR spectroscopy for monitoring drug release and functional tumor parameters, including oxygenation and redox status, in biologically relevant models [9,10,11].

Despite these advantages, EPR spectroscopy was, for many years, less frequently used in pharmaceutical laboratories than in specialized physical–chemical or bioanalytical settings. This was partly due to the cost and technical complexity of traditional spectrometers, the need for advanced expertise in spectral simulation and the fact that many pharmaceutical laboratories did not routinely investigate radical reactions. This situation is changing. Recent advances in instrumentation, including the development of benchtop instruments, together with improved computational tools and growing interest in oxidative stress and redox pharmacology, have made EPR spectroscopy more accessible to a wider scientific community. The technique is therefore no longer merely a specialized biophysical tool but an increasingly practical analytical approach for selected pharmaceutical problems.

A major advantage of EPR spectroscopy is that it can provide selective and non-destructive information in samples that may be transparent, colored, turbid, viscous, solid or biologically complex. Colorimetric antioxidant assays, for example, may be affected by sample color, opacity, precipitates or the absorbance of unrelated constituents, whereas EPR spectroscopy measures the radical signal itself. Consequently, the method is useful for the analysis of plant extracts, pharmaceutical preparations, lipid systems, polymeric carriers and biological fluids, particularly when direct radical detection is required [12,13,14].

EPR encompasses several experimental families. Continuous-wave (CW) EPR records the steady-state microwave absorption while the magnetic field is swept and is the principal focus of this review because it underpins most routine pharmaceutical applications described below. Pulsed EPR methods, including electron spin echo and double electron–electron resonance measurements, interrogate relaxation and nanometer-scale distances in the time domain, whereas electron–nuclear double resonance (ENDOR) resolves weak hyperfine interactions with nearby nuclei. These complementary techniques are important in structural biology and mechanistic studies, but a comprehensive treatment of them is beyond the present scope.

This article presents current applications and emerging opportunities of CW-EPR spectroscopy in pharmaceutical research, with emphasis on cases in which the method provides information that is difficult or impossible to obtain by other analytical approaches. Rather than offering an exhaustive catalogue of individual studies, this review focuses on fundamental concepts, representative experimental strategies and major areas of application, including radical detection, antioxidant activity, drug-induced toxicity, pharmaceutical stability, drug–membrane interactions, drug delivery systems, metallodrugs and microenvironmental measurements. Nevertheless, selected specific studies are discussed as illustrative examples to demonstrate the methodological value of EPR spectroscopy, typical spectral features and the type of mechanistic or microenvironmental information that can be obtained. In this way, EPR spectroscopy is presented as a methodological bridge between physical chemistry, biophysics, pharmaceutical technology, toxicology and pharmacology.

## 2. EPR Spectroscopy: Principles and Experimental Approaches

### 2.1. Fundamental Principles of EPR Spectroscopy

**Figure 1:**
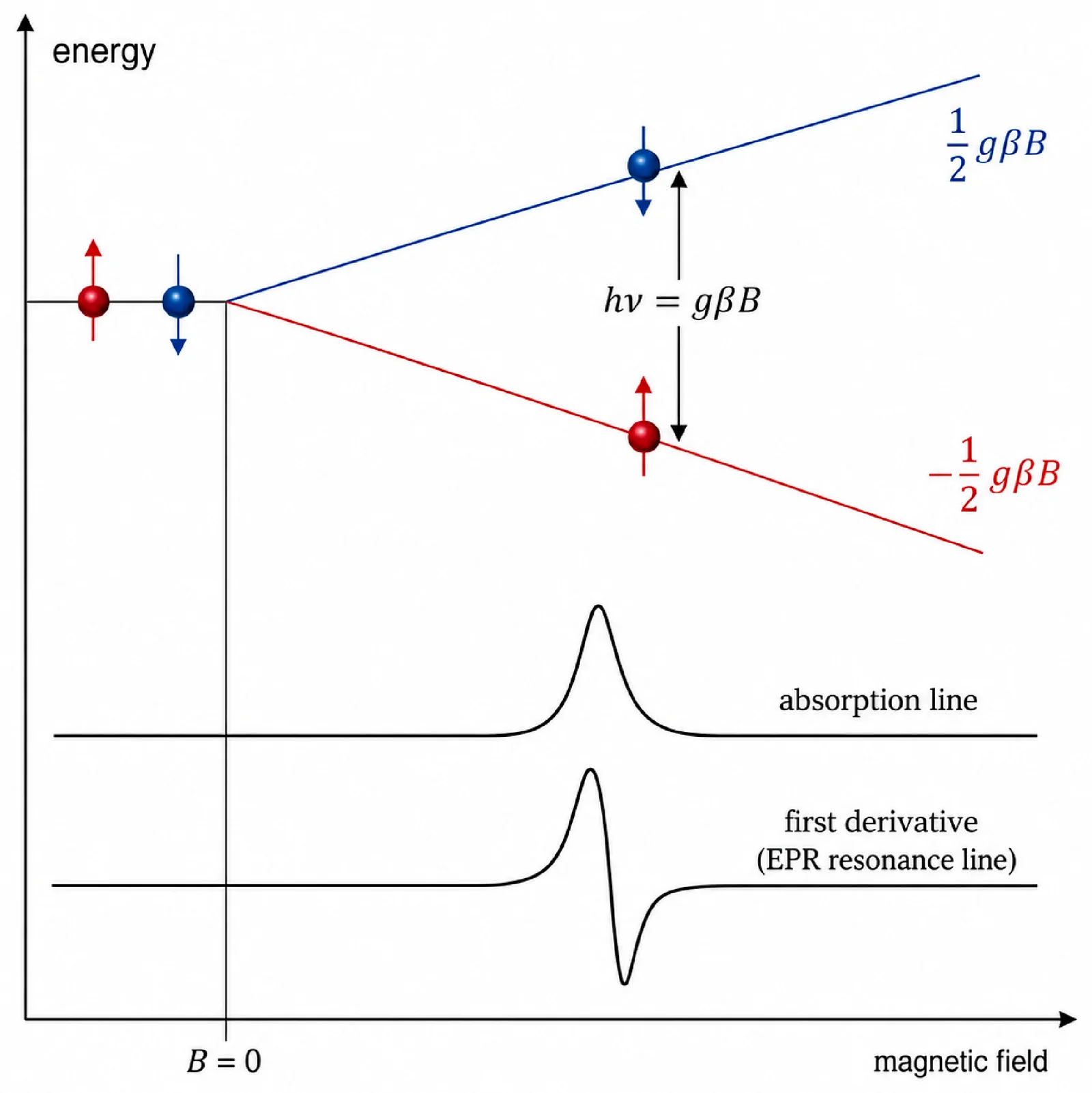
Basic principle of EPR spectroscopy: splitting of the energy levels of an unpaired electron in an external magnetic field (Zeeman effect) and absorption of microwave radiation.

EPR spectroscopy is based on the absorption of microwave radiation by paramagnetic species placed in an external magnetic field. A paramagnetic species contains an unpaired electron and therefore possesses a magnetic moment associated with electron spin. When such a species is exposed to a magnetic field, the energy level of the unpaired electron splits into two spin states, a phenomenon known as the Zeeman effect. Resonance occurs when the energy of the applied microwave radiation matches the energy difference between these two states, as schematically shown in Figure 1. This is described by the resonance condition:

Δ*E* = *hν* = *g β B*, (1)

where *h* is Planck’s constant, *ν* is the microwave frequency, *g* is the g-value, *β* is the Bohr magneton, and *B* is the magnetic-field strength [15]. The resonance condition represents the physical foundation of EPR measurement.

The relationship between microwave frequency, magnetic-field strength and the magnetic properties of the electron is described by the g-value. For a free electron, the g-value is approximately 2.0023, but in molecules, it may deviate from this value because the unpaired electron experiences orbital motion, local magnetic fields and the surrounding chemical environment. These deviations are analytically useful because they provide information about the location of the unpaired electron and the nature of the paramagnetic species. Radicals centered on carbon, nitrogen, oxygen or sulfur, as well as transition-metal complexes, may therefore be distinguished through characteristic g-values and their anisotropy.

**Figure 2:**
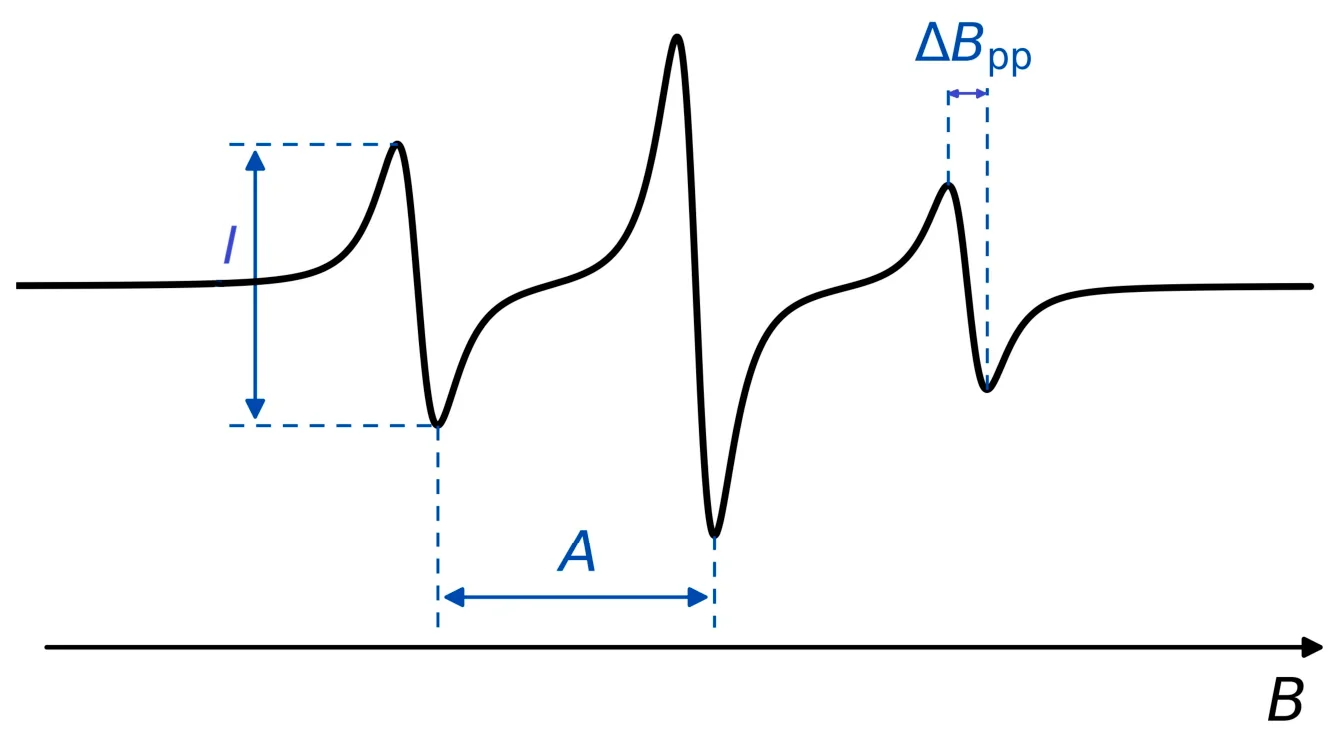
EPR spectrum of a typical nitroxide spin probe showing key spectral parameters: hyperfine splitting constant *A*, signal amplitude *I* and peak-to-peak linewidth Δ*B*_pp_.

In addition to the g-value, hyperfine splitting is one of the most important parameters of an EPR spectrum. Hyperfine splitting arises from the interaction between the unpaired electron and nuclei possessing a magnetic moment. The magnitude of this interaction is expressed by the hyperfine coupling constant, commonly denoted as *A*, although the symbol *a* is also used in the literature. For one nucleus with nuclear spin *I*, the number of hyperfine lines is given by 2*I* + 1. Therefore, interaction with a nitrogen nucleus of spin 1 often produces a triplet (2·1 + 1 = 3), whereas interaction with a proton of spin 1/2 produces a doublet (2·1/2 + 1 = 2). When the unpaired electron interacts with *N* equivalent nuclei of spin *I*, the spectrum is split into 2*NI* + 1 lines, with characteristic intensity ratios. The number of lines, their separation and their intensity ratios provide information on the number, identity and spatial arrangement of nuclei close to the unpaired electron. If an electron interacts with several non-equivalent nuclei, additional splitting occurs and the spectrum becomes a rich source of structural information [16,17,18]. Typical spectral parameters, including the hyperfine splitting constant, signal amplitude and linewidth, are illustrated in Figure 2.

EPR spectra are commonly recorded as the first derivative of the absorption curve. This presentation facilitates precise determination of resonance-line positions and linewidths, although it requires careful interpretation of signal intensity. The number of paramagnetic species is usually estimated by double integration of the spectrum. The first integration reconstructs the absorption curve, while the second integration provides an area proportional to the concentration of unpaired electrons. When appropriate calibration is performed, EPR spectroscopy can therefore be used not only qualitatively but also quantitatively.

The high sensitivity of EPR spectroscopy in comparison with nuclear magnetic resonance (NMR) spectroscopy arises from the much larger magnetic moment of the electron relative to atomic nuclei. For a narrow-line nitroxide under routine X-band CW conditions, practical detection is commonly in the low-micromolar range, with approximately 0.5–1.0 μM reported as an illustrative concentration limit [6]. This is not a universal detection limit: sensitivity worsens for broad or rapidly relaxing signals and improves with a high-Q resonator, optimized filling factor, signal averaging and a narrow linewidth. EPR spectroscopy does not provide a signal for diamagnetic molecules that do not contain unpaired electrons. This feature is both a limitation and an advantage: the technique is not universal for all molecular species, but it is highly selective for radicals, transition-metal ions and other paramagnetic systems.

In practical laboratory work, EPR spectroscopy is most frequently performed in the X-band region, at microwave frequencies of approximately 9–10 GHz. This range offers a favorable balance between sensitivity, instrumental feasibility and sample dimensions. Aqueous samples are often loaded in capillaries at volumes of roughly 10–100 μL or in conventional tubes at approximately 50–200 μL, depending on the resonator and the dielectric properties of the sample. A routine field-swept CW spectrum typically requires about 30 s to 10 min; repeated spectra acquired every few seconds to minutes can follow reaction or release kinetics, whereas extensive signal averaging or high-resolution scans require longer. These values are practical benchmarks rather than fixed limits. Other frequency ranges, including L-, S-, Q- and W-band instruments, are used for specialized purposes. Lower frequencies are advantageous for in vivo studies because microwave radiation penetrates more deeply into tissue, whereas higher frequencies can provide improved spectral resolution when the electronic structure and dynamics of paramagnetic centers are investigated.

The shape of an EPR spectrum is influenced by molecular motion, viscosity, temperature, polarity, spin concentration and the local organization of the sample. In low-viscosity liquids, small nitroxides can undergo nearly isotropic rotation with rotational correlation times (τ_c_) of approximately 10^−12^–10^−11^ s, producing narrow, motionally averaged lines. Restricted rotation in viscous phases, membranes and macromolecular assemblies increases τ_c_ into approximately the 10^−9^–10^−6^ s range and progressively reveals anisotropy and line broadening; the exact CW-EPR sensitivity window depends on the microwave frequency and label geometry [19,20]. Line broadening is not caused by motion alone. Through-space electron–electron dipole–dipole coupling increases as labeled sites become closer, while collision-mediated Heisenberg spin exchange becomes important at higher local spin concentrations. Both can change linewidths, line positions and apparent line multiplicity and can therefore imitate changes in mobility or oxygenation [21]. EPR lines are commonly described by Lorentzian, Gaussian or Voigt profiles. Lorentzian lines are associated mainly with homogeneous relaxation broadening, Gaussian lines with static distributions of local environments, and a Voigt profile with their convolution [22]. Mixed broadening is common in membranes, polymers, gels, nanoparticles and solid formulations; dilution series and spectral simulation help distinguish motion, heterogeneity and spin–spin interactions.

Reliable interpretation of EPR spectra often requires computer simulation. *EasySpin* is the most widely used and established software package for advanced EPR spectral simulation and analysis [23]. Experimental spectra are compared with simulated spectra by varying g-values, hyperfine coupling constants, linewidths, relative intensities and the contributions of multiple radical species. In parallel, more accessible tools such as *VisualEPR* are being developed as free online platforms for beginners and rapid spectral visualization [24]. This approach is indispensable in complex systems in which several paramagnetic species coexist or overlap. Software-assisted simulation has greatly expanded the practical applicability of EPR spectroscopy by allowing more robust assignment of signals in chemically and biologically complex samples.

For pharmaceutical applications, the fundamental EPR parameters have direct analytical meaning. The g-value assists in identifying radical or metal-centered species; hyperfine splitting provides structural information; linewidth reflects relaxation, molecular motion and interactions with oxygen or other paramagnets; and signal intensity enables quantification. These parameters form the conceptual basis for the applications discussed in the following sections, including radical detection, membrane analysis, drug release studies, metallodrug characterization and EPR oximetry.

When the pharmaceutical target is diamagnetic or a radical is too short-lived for direct observation, three complementary indirect CW-EPR strategies are used. Spin trapping converts a transient radical into a longer-lived paramagnetic adduct and is therefore primarily a radical-identification method. Spin labeling covalently attaches a stable radical to a defined molecule or site and reports on that site’s structure, dynamics or interactions. Spin-probe analysis introduces a stable radical without site-specific covalent attachment and uses its spectrum to map the properties of the surrounding microenvironment, including mobility, polarity, viscosity, oxygenation and pH. Keeping these strategies conceptually separate is important because their controls and the meaning of their spectra differ.

### 2.2. Spin Trapping Technique

Spin trapping is an important EPR approach for detecting short-lived radical species that are usually too reactive to be observed directly. The method is based on the reaction of a transient radical with a diamagnetic spin trap, producing a more stable radical adduct that can be detected by EPR spectroscopy. In this way, a radical that would otherwise decay before direct measurement is converted into a spectroscopically accessible species [5,25].

**Figure 3:**
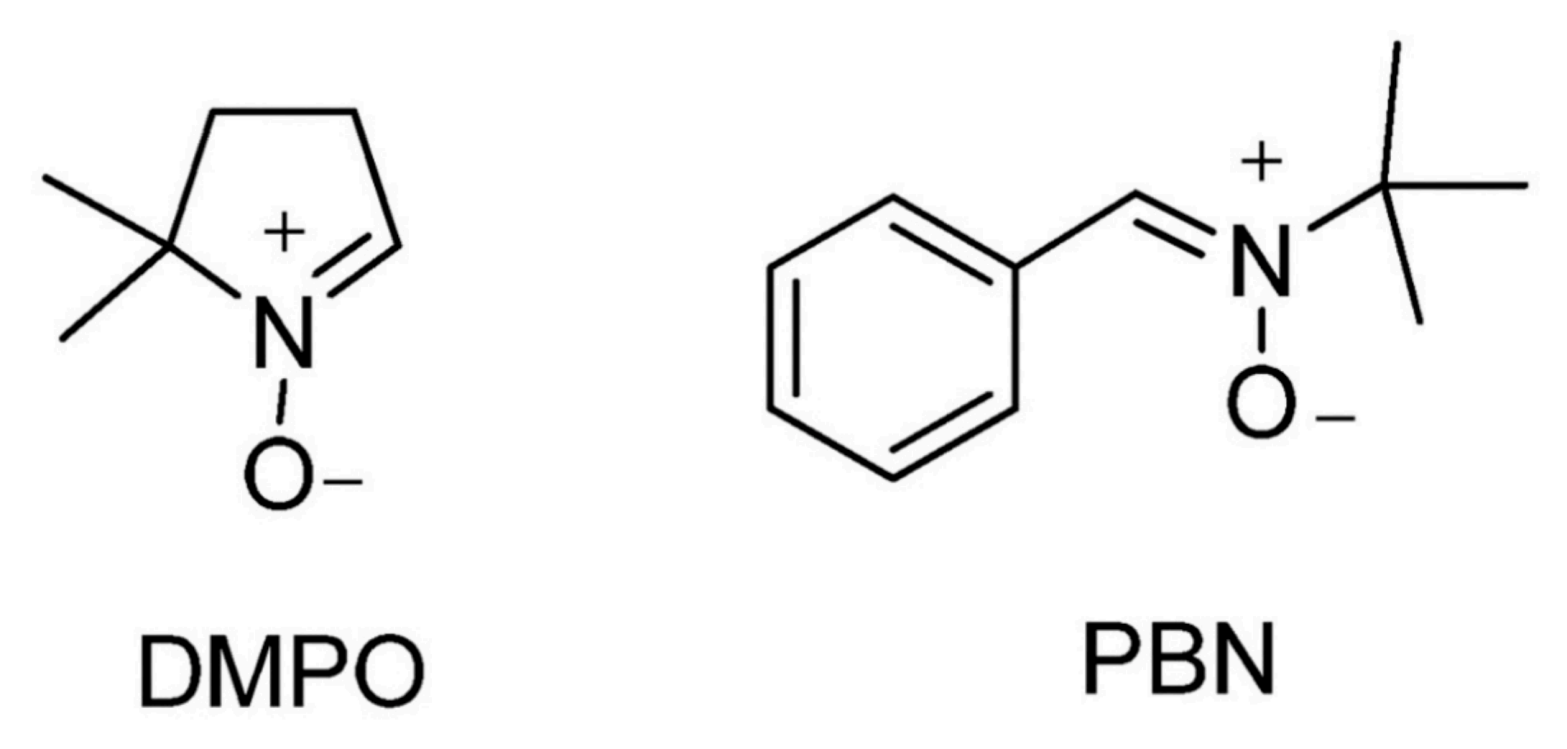
Chemical structures of the classical spin traps DMPO and PBN, which are commonly used for EPR detection of short-lived radical species.

The choice of spin trap is crucial for the success of the experiment. Nitrones and nitroso compounds are among the most widely used spin traps, with 5,5-dimethyl-1-pyrroline N-oxide (DMPO) and N-tert-butyl-alpha-phenylnitrone (PBN) being classical examples. Their chemical structures are shown in Figure 3. These compounds react with reactive oxygen species (ROS), carbon-centered radicals, nitrogen-centered radicals and other transient species produced by chemical reactions, irradiation or enzymatic processes. The resulting EPR spectra provide information not only on radical presence but also on radical identity and mechanism of formation.

In a typical spin-trapping experiment, the radical source, spin trap and experimental conditions must be carefully selected. Radical generation may be initiated chemically, photochemically, enzymatically or by ionizing radiation [5,25]. The resulting spin-adduct spectrum is then interpreted using its characteristic g-values and hyperfine coupling constants. These parameters serve as a spectral fingerprint of the trapped radical and can be compared with spectral libraries or refined by computer simulation.

**Figure 4:**
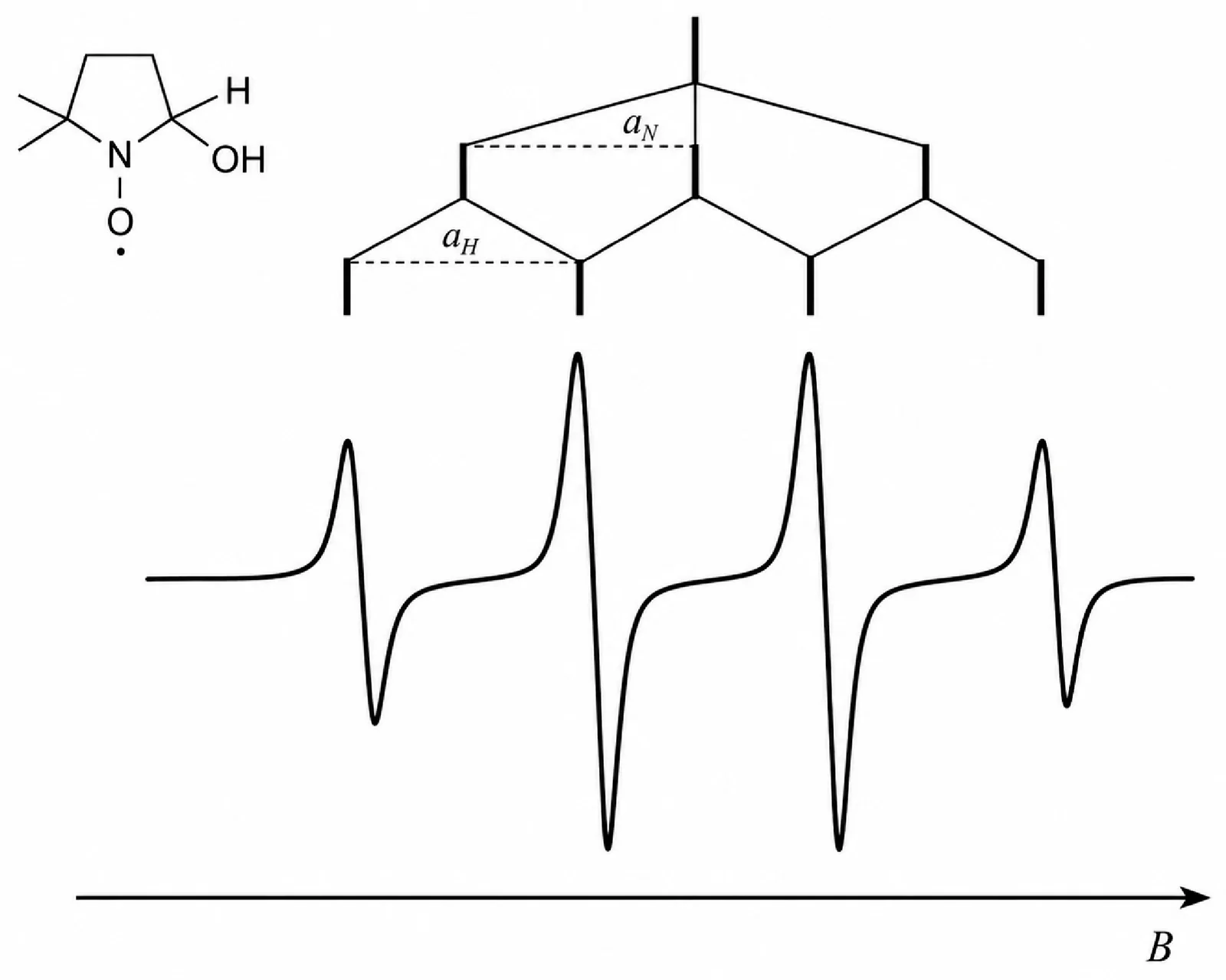
Simulated CW-EPR spectrum of the DMPO–OH spin adduct showing the characteristic four-line pattern with an intensity ratio of 1:2:2:1. VisualEPR simulation parameters: isotropic g-value g_iso_ = 2.0057; nitrogen and β-proton hyperfine coupling constants a_N_ = a_H_ = 14.9 G; Lorentzian peak-to-peak linewidth ΔB_pp_ = 1.0 G.

Common spin adducts differ in both stability and spectral appearance. DMPO-OH, produced by trapping hydroxyl radicals, is typically recognized by a four-line EPR pattern with a 1:2:2:1 intensity ratio, as illustrated in Figure 4. DMPO-OOH, the superoxide adduct, is less stable and gives a more complex spectrum; its interpretation requires caution because secondary reactions may produce DMPO-OH-like signals [5,26]. DMPO adducts of carbon-centered radicals show spectra that depend strongly on radical structure and associated hyperfine coupling constants, whereas sulfur-centered adducts may be complicated by rapid transformation or decay [27,28,29]. PBN is widely used for carbon-centered radicals and often gives relatively simple nitroxide spectra dominated by nitrogen and β-hydrogen hyperfine splitting [5,6]. Thus, the identity of a trapped radical is inferred not only from the presence of an EPR signal but from the complete spectral pattern, including the g-value, hyperfine coupling constants, linewidth and comparison with simulated spectra.

The short lifetime of DMPO–OOH, typically on the order of one minute under aqueous conditions, motivated the development of second-generation cyclic nitrone traps. 5-(Diethoxyphosphoryl)-5-methyl-1-pyrroline N-oxide (DEPMPO) and 5-tert-butoxycarbonyl-5-methyl-1-pyrroline N-oxide (BMPO) form superoxide adducts that commonly persist for several to tens of minutes, depending on the pH, solvent, temperature and reducing capacity [25,26,30]. Their longer observation window improves superoxide assignment in chemical and cellular experiments. Nevertheless, adduct stability alone does not determine performance: the radical-trapping rate, cell permeability, bioreduction and conversion of hydroperoxyl adducts into hydroxyl-like products must still be evaluated with controls.

Spin trapping has broad relevance for pharmaceutical research. It can reveal whether a drug generates radicals during metabolism, whether a pharmaceutical preparation produces radicals after exposure to light or heat, or whether a candidate antioxidant suppresses radical formation [4,5,6]. It is especially valuable in mechanistic toxicology, where the detection of radical intermediates may clarify why a compound damages membranes, proteins or DNA [6]. In formulation science, spin trapping can be applied to study oxidative degradation pathways in excipients, polymers, lipids and dispersed systems [31,32,33].

Spin trapping is also useful in photochemical and phototoxicity studies. Many pharmaceutical compounds, excipients and UV filters can generate radical intermediates after exposure to light, especially in the presence of oxygen or redox-active impurities. EPR spin trapping can help identify whether irradiation produces oxygen-centered, carbon-centered or other radical species, which is important for understanding photodegradation pathways and improving the photostability of pharmaceutical preparations [5,34].

Another important application is the study of radical processes in biological and cellular systems. In such experiments, spin trapping can be used to detect reactive oxygen and nitrogen species generated during oxidative stress, inflammation, or drug metabolism. This is particularly relevant in pharmacology and toxicology because radical formation may contribute both to therapeutic effects and to adverse reactions. However, interpretation in biological samples requires caution because spin adducts may be affected by enzymatic reactions, reductants, oxygen concentration and cellular uptake [12,35].

**Figure 5:**
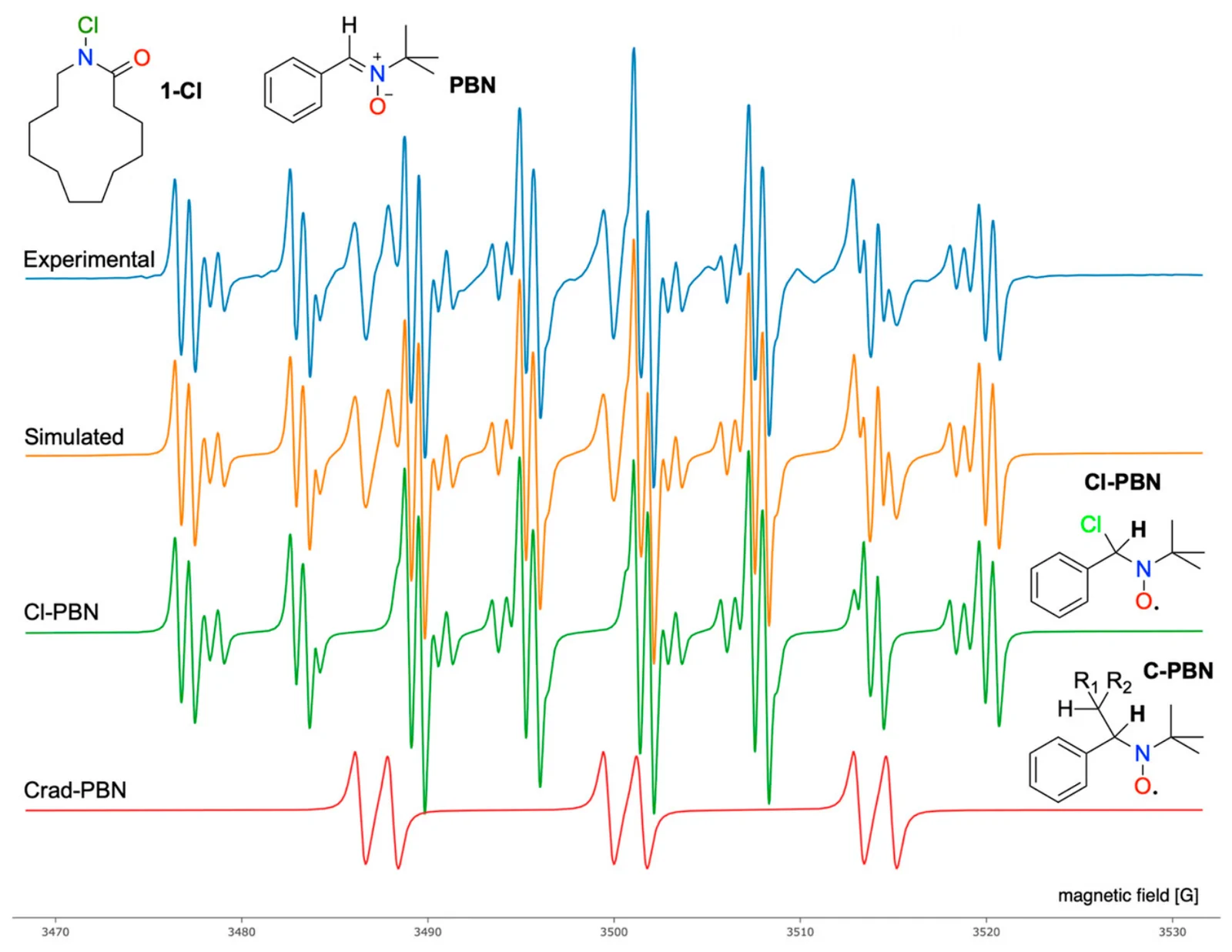
EPR spectra of spin-trapped radical intermediates generated upon UV irradiation of 1-Cl at 370 nm. The experimental spectrum is shown in blue; the simulated Crad-PBN and Cl-PBN spin adducts are shown in red and green, respectively; and the total simulated spectrum, obtained as their sum, is shown in orange [18].

The method also has value in synthetic and medicinal chemistry, where radical-mediated transformations can support the late-stage functionalization of drug-like molecules. A representative example is the Hofmann–Löffler–Freytag-type rearrangement of N-chlorinated laurolactam, 1-chloroazacyclotridecan-2-one (1-Cl). This transformation is relevant because the conversion of macrocyclic lactams into more rigid bicyclic structures may improve the hydrolytic and metabolic stability of peptide-based drug candidates. Upon UV irradiation at 370 nm, radical intermediates were trapped with PBN and characterized by EPR spectroscopy, as shown in Figure 5. The experimental spectrum closely matches the total simulated spectrum, obtained as the sum of the Cl-PBN and Crad-PBN spin adducts, supporting the assignment of chlorine-centered and carbon-centered radical intermediates. Together with NMR analysis and quantum chemical calculations, these results clarify the radical mechanism and support the pharmaceutical relevance of this rearrangement [16,17,18]. Spectral fitting and visualization were performed using *VisualEPR*.

Despite its usefulness, spin trapping requires critical interpretation. Spin adducts may decompose, rearrange or be formed indirectly. Different radicals may also react with the same spin trap at different rates, while oxygen, pH, metal ions and solvent composition may alter the observed spectrum [27,28,29]. Therefore, spin-trapping experiments should be accompanied by appropriate controls, kinetic measurements and, when possible, comparison with simulated spectra. Used carefully, the technique provides a powerful window into radical chemistry in pharmaceutical and biological systems.

### 2.3. Spin Labeling Technique

Spin labeling is used to investigate the structure, dynamics and interactions of biomolecules, membranes, polymers and other complex systems. In contrast to spin trapping, spin labeling deliberately and usually covalently introduces a stable paramagnetic group at a defined position in an otherwise EPR-silent molecule. The selected position can then be monitored by CW-EPR [6,20,36]. Nitroxide labels are most common; examples include maleimide- or methanethiosulfonate-based labels attached to engineered cysteine residues and doxyl groups incorporated at defined positions in lipid acyl chains. The resulting spectrum reports on the mobility, accessibility and interactions of the labeled site rather than on the average bulk environment.

The distinction between spin traps and spin labels is analytically important. A spin trap is initially EPR-silent and becomes paramagnetic only after it reacts with a transient radical; the adduct spectrum is interpreted to identify the radical. A spin label is already paramagnetic and is attached to a selected molecular site; changes in its spectrum are interpreted as changes in that site’s mobility, conformation, accessibility or intermolecular proximity [20,36]. Consequently, the chemical stability and site specificity of the label must be confirmed, and the labeled construct should be compared with its unlabeled counterpart to verify that labeling has not changed the relevant function.

Spin labeling is particularly valuable for biological membranes. The standard depth series comprises 5-, 12-, and 16-doxyl stearic acid (5-, 12-, and 16-DSA; also abbreviated 5-, 12-, and 16-DS). The nitroxide is positioned progressively farther from the polar head-group region, so 5-DS reports mainly on the interfacial region, 12-DS on the intermediate acyl-chain region and 16-DS on the hydrophobic core. Comparing the depth-resolved spectra before and after drug addition shows whether a compound selectively disorders or rigidifies a bilayer region or changes lipid–protein interactions. This approach is useful for amphiphilic drugs, local anesthetics, beta-blockers, non-steroidal anti-inflammatory drugs and membrane-active antimicrobial agents [37,38].

Spin labeling also supports structural biology and protein research. Site-directed spin labeling, in which a nitroxide label is introduced at a selected amino acid residue, allows investigation of conformational changes, accessibility and molecular interactions. In routine applications, CW-EPR analyzes the line shape, mobility and local environment of one labeled site. Pulsed EPR techniques use short microwave pulses to detect spin interactions in the time domain and, with pairs of labels, can measure nanometer-scale distances that provide structural constraints complementary to crystallography, NMR spectroscopy and cryo-electron microscopy [20,39]. Although these applications are more common in biophysics, they are increasingly relevant to membrane proteins, transporters and protein–drug interactions [37,38].

Overall, spin labeling converts a selected molecular position into a site-specific EPR reporter. It is therefore most informative when the pharmaceutical question concerns a defined residue, lipid depth or conjugated drug/carrier component. Non-covalent environmental reporters are treated separately in the following section.

### 2.4. Spin-Probe Technique

**Figure 6:**
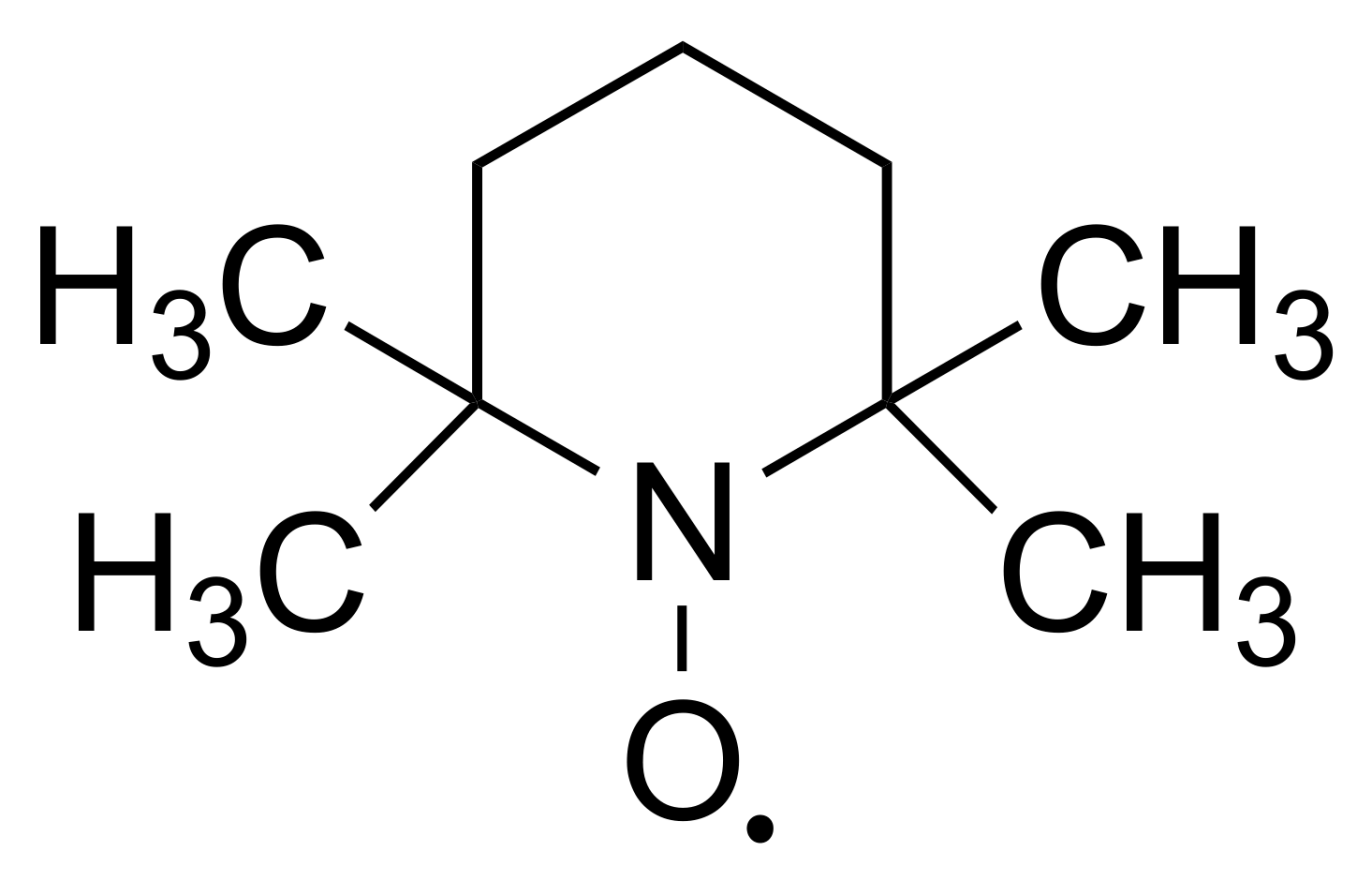
Chemical structure of TEMPO, a stable nitroxide probe widely used for non-covalent spin-probe measurements and as a building block for covalent spin labels.

A spin probe is a stable paramagnetic molecule added to a sample without site-specific covalent attachment. Its distribution and spectrum report on the local environment that it samples. TEMPO, TEMPOL and PROXYL derivatives are widely used because their three-line ^14^N nitroxide spectra respond to rotational mobility, solvent polarity, hydrogen bonding, oxygen and redox conditions. Unlike a spin label, a probe may partition dynamically among water, lipid, polymer and carrier domains; multiple spectral components can therefore quantify coexisting environments. The TEMPO probe is shown in Figure 6 [18,19,40].

**Figure 7:**
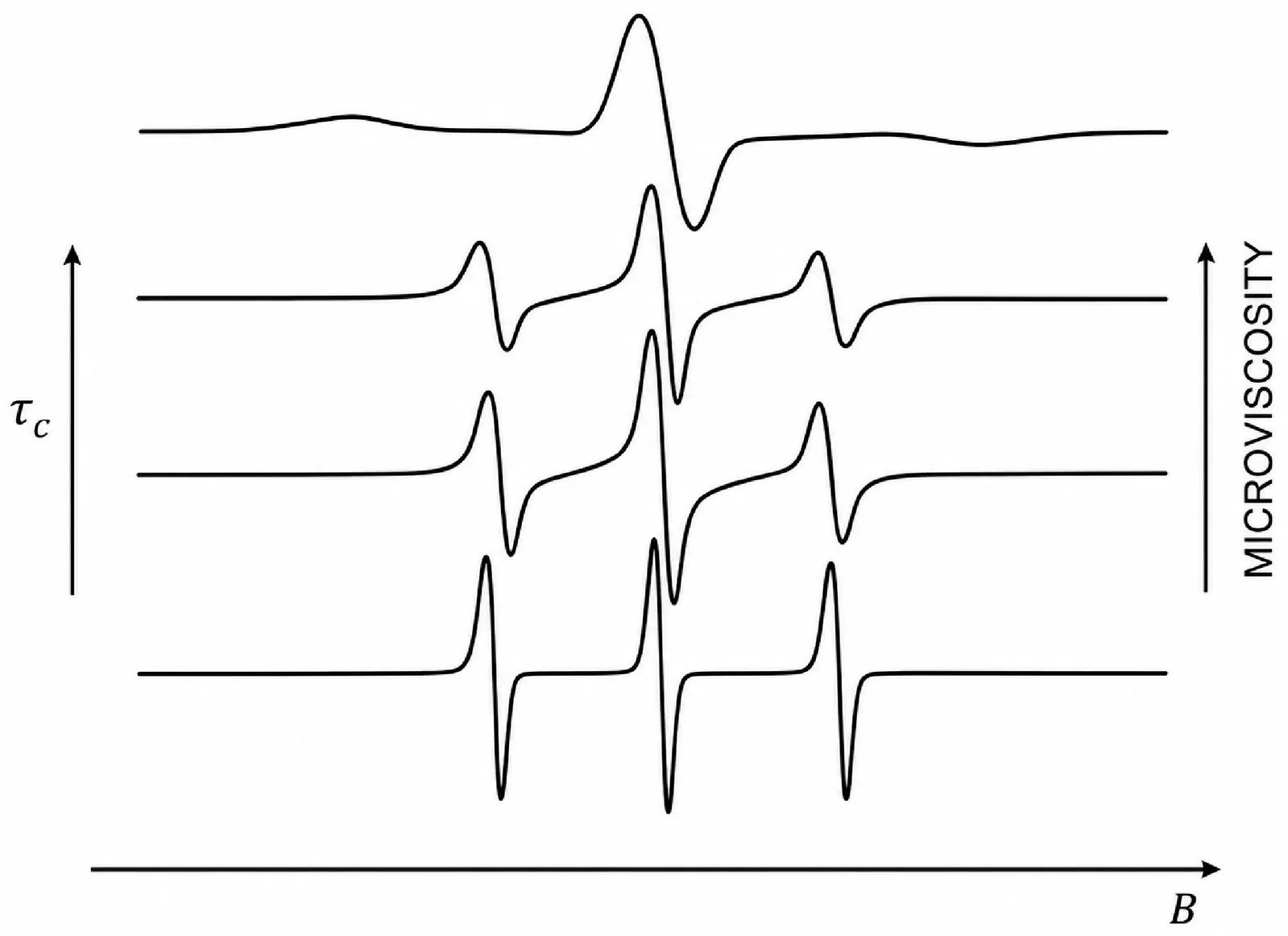
Typical CW-EPR spectral changes of a nitroxide spin probe in environments with different microviscosities. Increasing rotational correlation time indicates slower motion, greater microviscosity and broader, increasingly anisotropic lines.

Probe mobility is commonly expressed through the rotational correlation time τ_c_. In a low-viscosity liquid, rapid tumbling averages anisotropic g and hyperfine interactions and yields three narrow, nearly symmetric lines; small nitroxides often have τ_c_ values near 10^−12^–10^−11^ s. As the viscosity or steric confinement increases, τ_c_ rises, the outer lines broaden and the line-height ratios become unequal. In the fast-motion regime, τ_c_ can be estimated from the linewidth and line-height ratios, whereas slow-motion spectra generally require simulation. Figure 7 illustrates these changes. Because temperature also changes τ_c_, viscosity comparisons require controlled temperature or calibration with standards [18,19].

In pharmaceutical technology, non-covalent spin probes characterize liposomes, microemulsions, polymeric nanoparticles and hydrogels. A hydrophilic probe reports mainly on aqueous compartments, while a lipophilic probe samples interfaces and hydrophobic domains. Hyperfine splitting can distinguish environments of different polarity, and the relative areas of mobile and immobilized spectral components can follow hydration, leakage, swelling or matrix degradation. These measurements remain possible in opaque and concentrated samples for which optical scattering or absorbance complicates fluorescence analysis [9,19,40].

Thus, spin probes are environmental reporters rather than site-specific labels. Their interpretation requires attention to probe partitioning, concentration, reduction and possible perturbation of the system; when these factors are controlled, they provide local information that cannot be inferred from bulk viscosity or composition alone.

## 3. Applications of EPR Spectroscopy in Pharmaceutical Research

EPR spectroscopy has a distinct role in pharmaceutical research because it provides direct access to paramagnetic species. This is particularly important when the process under investigation involves free radicals, radical intermediates, transition-metal centers, oxygen-sensitive probes or spin-labeled molecules. In this context, EPR spectroscopy should not be viewed as a general replacement for chromatographic, optical or mass spectrometric techniques. Its value lies in questions for which unpaired electrons, redox chemistry, oxidative degradation or local molecular environments are central.

**Figure 8:**
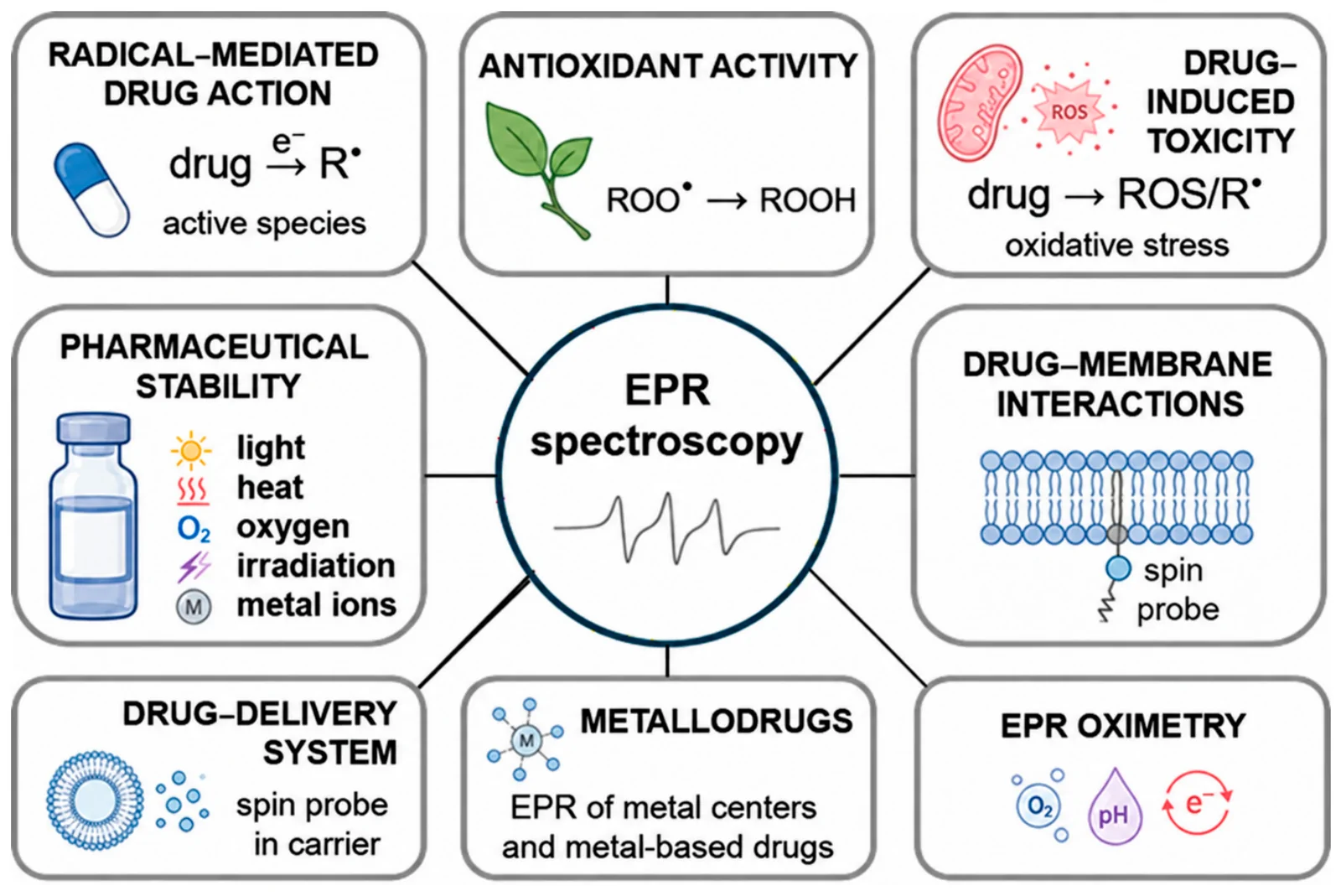
Overview of major applications of EPR spectroscopy in pharmaceutical research.

In pharmaceutical research, EPR spectroscopy can be applied at several stages of drug discovery, development and quality assessment. In early screening, it can help identify compounds with radical-scavenging activity or detect radical intermediates generated during drug activation. During formulation development, it can be used to examine oxidative stability, drug–membrane interactions, carrier structure and drug release. In biological models, EPR-based methods can monitor oxygenation, pH, redox status and other features of the tissue microenvironment. These major application areas are summarized in Figure 8. Together, they place EPR spectroscopy at the interface of pharmaceutical chemistry, biophysics, toxicology, formulation science and drug delivery research [4,6,40].

### 3.1. Free Radicals, Antioxidant Activity and Redox-Active Drugs

Free radicals and related reactive species are involved in many physiological and pharmaceutical processes. They can damage lipids, proteins, nucleic acids and biological membranes, but they also participate in redox signaling, enzyme activity, immune responses and drug metabolism. The distinction between physiological redox signaling and excessive oxidative damage is important because radical formation is not always harmful. In pharmaceutical research, this makes direct radical detection relevant for antioxidant screening, mechanistic pharmacology and the study of redox-active drugs [41,42,43].

EPR spectroscopy is especially useful for evaluating antioxidant activity because it measures the radical signal directly. Colorimetric UV antioxidant assays may be influenced by sample color, turbidity, precipitates or unrelated absorbance. EPR spectroscopy is less affected by these limitations and can therefore be applied to optically complex samples. This is important for plant extracts, natural products, polyphenol-rich mixtures, lipid systems and concentrated pharmaceutical samples [4,6,13,14].

**Figure 9:**
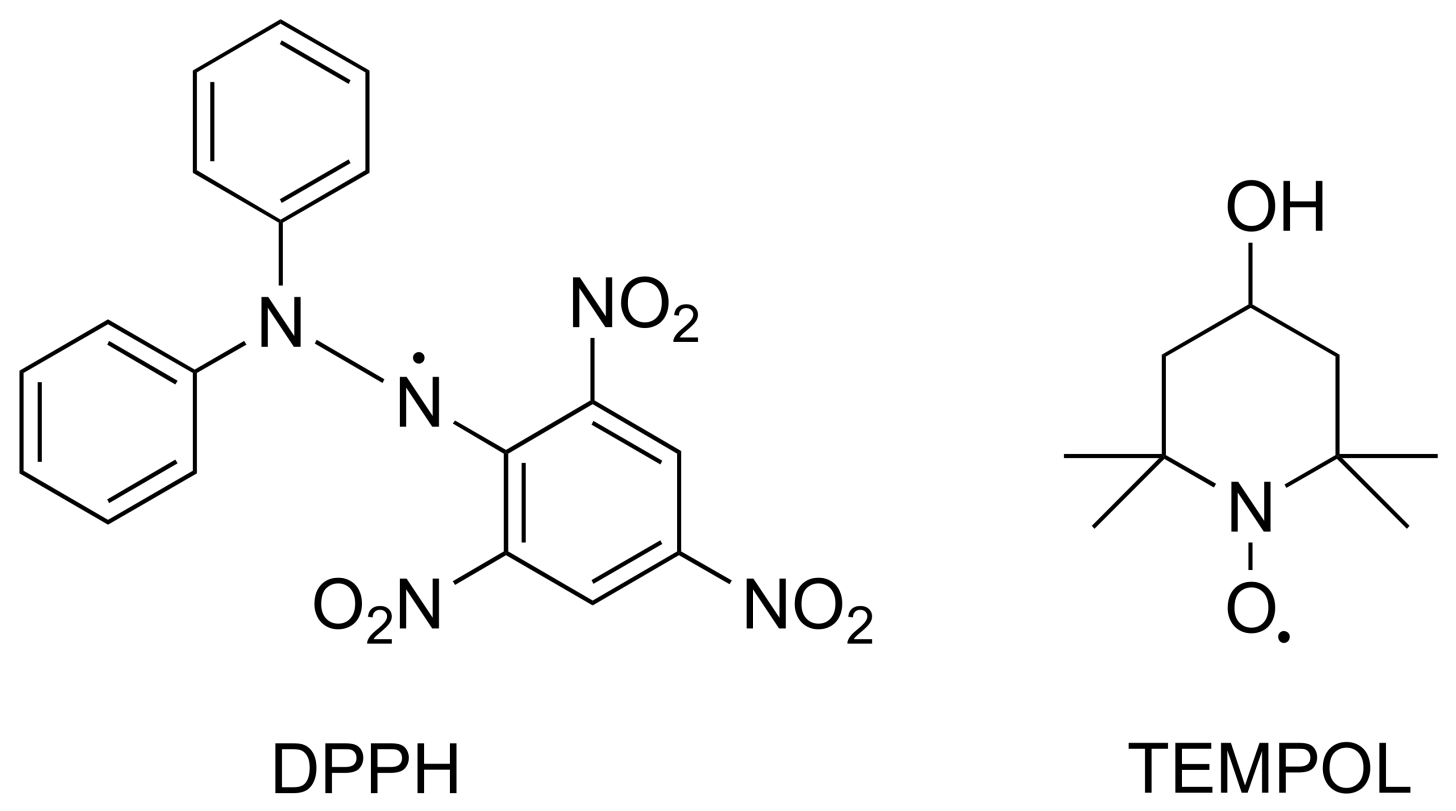
Chemical structures of DPPH and TEMPOL, which are representative stable radicals used in EPR-based antioxidant assays.

Stable radicals are commonly used as reference systems in EPR-based antioxidant assays. Among them, 2,2-diphenyl-1-picrylhydrazyl (DPPH) and 4-hydroxy-2,2,6,6-tetramethylpiperidine-1-oxyl (TEMPOL) are frequently applied because they give well-defined EPR signals that decrease after reaction with radical-scavenging compounds. In an EPR-DPPH assay, the rate and extent of signal decay can be used to estimate antioxidant capacity. The double integral of the EPR spectrum corresponds to the area under the absorption curve and is proportional to the amount of remaining radical. This makes the approach useful not only for detecting antioxidant activity but also for comparing antioxidant efficiency among different samples. Representative structures of DPPH and TEMPOL are shown in Figure 9.

Although many examples of EPR-based antioxidant testing come from food chemistry, they are still relevant to pharmaceutical research. Natural products, nutraceuticals, dermatological preparations and dietary supplements often contain antioxidants from plant, wine, beer, honey or oil matrices. These systems are chemically complex and may contain pigments, polyphenols, lipids and suspended material. EPR-DPPH studies on beer and wine, for example, demonstrate how antioxidant capacity can be measured in optically complex samples and related to chemical composition [44,45]. Similar reasoning applies to honey and vegetable oils, which are relevant as sources of natural antioxidants and as models for lipid-rich pharmaceutical and cosmetic systems [33,46,47].

**Figure 10:**
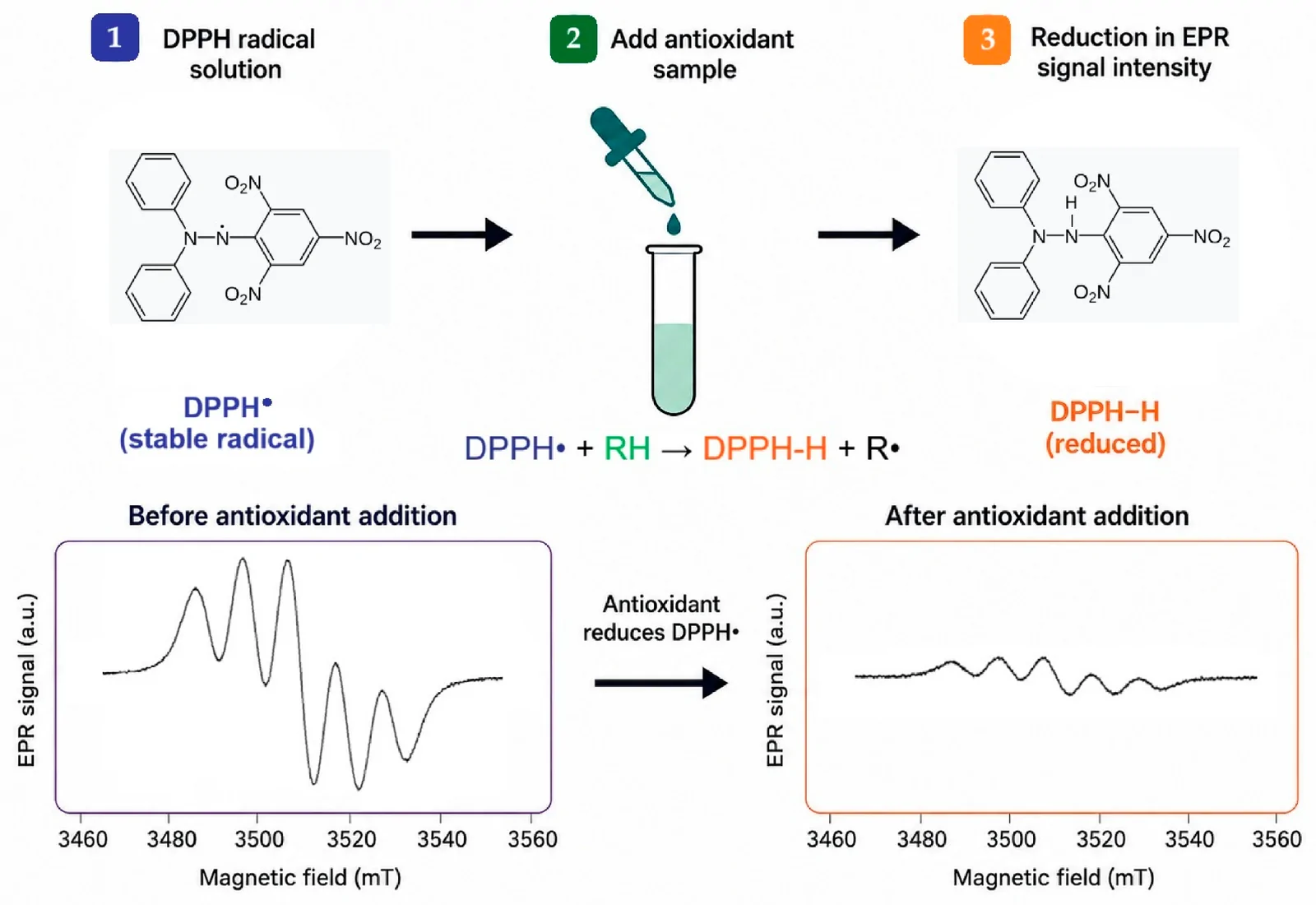
Principle of the EPR-DPPH radical-scavenging assay. DPPH^•^ gives a characteristic five-line EPR spectrum, while its reduction to DPPH-H after antioxidant addition leads to a decrease in signal intensity.

A typical EPR-DPPH radical-scavenging experiment is illustrated in Figure 10. DPPH gives a characteristic EPR spectrum composed of five lines, which arise from hyperfine interaction of the unpaired electron with the two equivalent nitrogen nuclei of the hydrazyl radical system. After addition of an antioxidant, the DPPH radical is reduced to the EPR-silent DPPH-H form, leading to a decrease in signal intensity. This progressive signal decay reflects the reduction of DPPH radicals by antioxidant compounds and can be used to evaluate radical-scavenging activity.

Lipid-rich systems are particularly important because oxidation can begin with short-lived radical species that are difficult to detect by conventional methods. EPR spin trapping can capture early lipid-derived radicals and provide information before secondary oxidation products become dominant. This is relevant for edible oils but also for lipid excipients, liposomes, lipid nanoparticles, emulsions and semisolid pharmaceutical preparations. Studies on vegetable oils and emulsified systems show that EPR spectroscopy can be used to evaluate oxidative resistance and the protective effect of antioxidants under stress conditions [48,49].

EPR spectroscopy is also useful for studying redox-active drugs and radical intermediates involved in drug action. Some anticancer, antimalarial and nitric oxide-releasing drugs act, at least in part, through radical or paramagnetic intermediates. Direct EPR spectroscopy can monitor stable paramagnetic species, whereas spin trapping enables the detection of short-lived radical intermediates. This helps distinguish whether a drug acts through electron transfer, radical generation, metal-mediated redox cycling or reactions with oxygen-derived species [5,6,12].

One example is the study of radical anions generated by antitumor compounds such as iminoquinone derivatives. EPR spectroscopy can detect radical intermediates formed during reduction reactions and thereby support mechanistic interpretation of cytotoxic activity [50]. This is important because radical generation may contribute to the therapeutic effect but may also participate in unwanted toxicity.

Antimalarial drugs provide another example. Artemisinin and related peroxide-containing drugs can generate carbon-centered radical intermediates after activation, especially in iron-containing systems. EPR spin trapping with DMPO or related traps can help detect these intermediates and clarify radical pathways involved in antimalarial activity [51,52]. Related EPR-based approaches can also be applied to redox-active drug candidates beyond classical antimalarials. For example, harmicenes, which are harmine–ferrocene hybrids with antiglioblastoma potential, were recently evaluated using the EPR-DPPH assay to assess their radical-scavenging behavior as part of a broader investigation of their biological activity [53].

### 3.2. Radical-Mediated Toxicity, Pharmaceutical Stability and Membrane Interactions

Free radical formation is relevant not only to drug activity but also to drug-induced toxicity. Reactive intermediates generated during metabolism may damage membranes, proteins and DNA. EPR spectroscopy can help detect these intermediates directly or through spin trapping, making it useful in mechanistic toxicology. This is particularly important when oxidative stress, redox cycling, lipid peroxidation or phototoxicity is suspected [14,29,54].

Anthracycline-induced cardiotoxicity is a well-known example of radical-associated drug toxicity. Redox cycling of anthracycline quinones can contribute to the formation of ROS and oxidative damage in cardiac tissue. Similar radical-mediated processes have been investigated for antimalarial drugs, non-steroidal anti-inflammatory drugs, quinone-containing compounds and photosensitive pharmaceutical substances. EPR spectroscopy contributes to such studies by providing direct or indirect evidence of radical formation, complementing biochemical assays of lipid peroxidation, enzyme inhibition or oxidative DNA damage [51,54].

**Figure 11:**
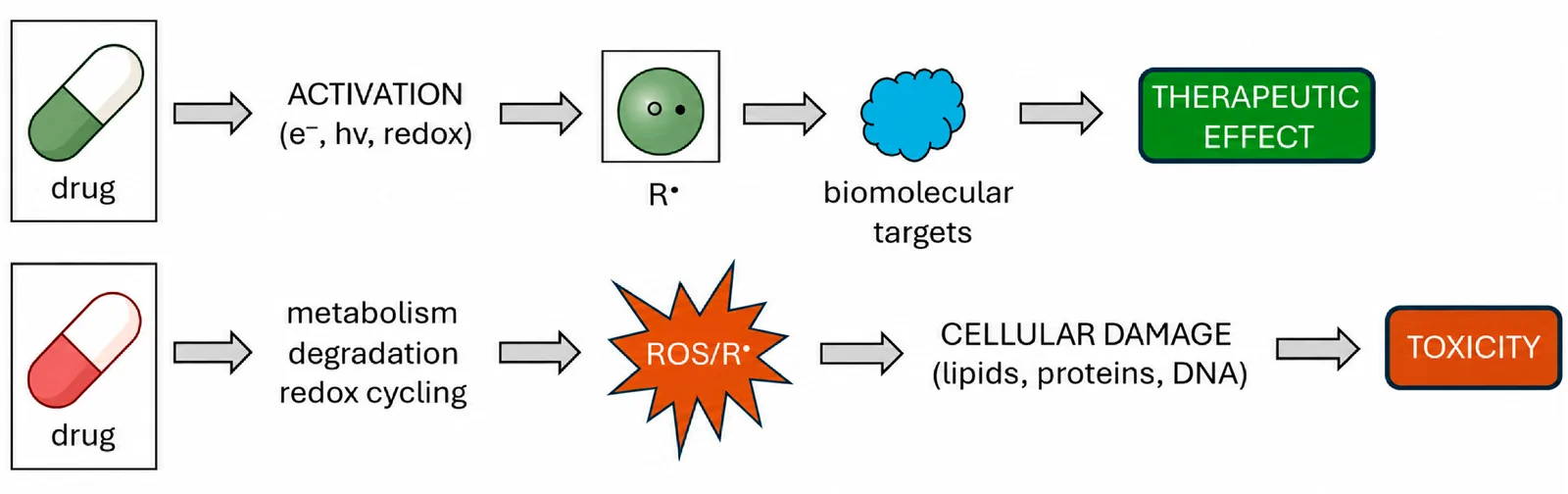
Dual role of radical formation in drug action and toxicity. Drug activation may generate radical intermediates that contribute to therapeutic effects, whereas metabolism, degradation or redox cycling may produce ROS or drug-derived radicals that damage lipids, proteins and DNA and contribute to toxicity.

The dual role of radical formation in drug action and toxicity is summarized in Figure 11. In some cases, drug activation by electron transfer, light or redox processes generates radical intermediates that interact with biomolecular targets and contribute to the therapeutic effect. In other cases, metabolism, degradation or redox cycling produces ROS or drug-derived radicals that damage lipids, proteins and DNA, ultimately contributing to toxicity. This distinction is important because EPR spectroscopy can help determine whether radical formation is part of the desired pharmacological mechanism or an unwanted degradation- or toxicity-related pathway.

Non-steroidal anti-inflammatory drugs also illustrate the value of EPR spectroscopy in toxicity studies. In model systems, piroxicam can participate in reactions leading to glutathionyl radical formation. The detection of such species provides evidence for redox processes that may contribute to tissue injury or altered antioxidant defense. EPR spectra obtained under different reaction conditions can help identify which components of the system are required for radical formation [6,55].

Pharmaceutical stability is one of the most practical areas in which EPR spectroscopy can provide unique information. Free radicals may be generated during photodegradation, autoxidation, metal-catalyzed oxidation, irradiation, thermal treatment, mechanical stress or sterilization. These processes can affect active pharmaceutical ingredients, excipients, polymers, packaging materials and final dosage forms. Because EPR spectroscopy detects radicals and paramagnetic transition-metal impurities directly, it can reveal early degradation pathways in active substances, excipients, surfactants and final dosage forms that may not be evident from conventional chromatographic or optical analysis [7,47,56].

This application is also emphasized in industrial and benchtop EPR contexts. EPR spectroscopy has been presented as a tool for detecting paramagnetic impurities, monitoring radical-generating degradation processes, evaluating stress testing, assessing sterilization-induced radicals and supporting shelf-life prediction. These uses are relevant to quality control because radicals or transition-metal impurities may reduce potency, shorten shelf life or contribute to toxicity [57].

Stress testing is a useful example. The exposure of pharmaceutical materials to light, heat, oxygen, moisture or chemical oxidants can accelerate degradation and reveal radical-forming pathways. EPR spectroscopy can then be used to compare formulations, evaluate the protective role of antioxidants and identify conditions that should be avoided during manufacturing, transport or storage. The increasing availability of compact benchtop instruments may make such applications more realistic for applied pharmaceutical laboratories [4,6].

Protein and peptide therapeutics add a current pharmaceutical priority. Oxidation can generate amino-acid-centered radicals, including tyrosyl (Tyr), tryptophanyl (Trp) and cysteinyl/thiyl (Cys) radicals. These intermediates can initiate dimerization and cross-linking, backbone cleavage, aggregation or loss of biological activity and may therefore affect potency and immunogenicity. Because most are short-lived, their assignment commonly requires rapid freeze-quenching, spin trapping or detection of secondary radical products. EPR evidence for Tyr- and Cys-centered chemistry can help connect formulation stressors such as light, peroxides or trace metals with specific degradation pathways in therapeutic proteins [7,14,55].

Drug–membrane interactions provide a natural extension of these toxicity- and stability-related applications. Biological membranes are frequent targets of oxidative damage, but they are also key determinants of drug absorption, distribution, pharmacological action and adverse effects. Many drugs interact with lipid bilayers by partitioning into membranes, changing lipid packing or altering membrane dynamics. These effects may influence membrane permeability, receptor function, transport processes and cellular toxicity. EPR spin labeling is well suited for such studies because spin-labeled lipids and fatty acids can report on specific regions of the membrane [37,58].

The standard doxyl-stearic-acid series, that is, 5-, 12-, and 16-DS, probe progressively deeper positions in the lipid bilayer. The 5-DS spectrum is dominated by the interfacial region near the polar head groups, 12-DS reports on the middle of the acyl chain, and 16-DS reports on the hydrophobic core. Drug-induced changes in the line shape, order parameters, linewidth and rotational correlation time therefore reveal not only whether a membrane is perturbed but also where the perturbation is greatest [37,38,58].

This approach has been applied to amphiphilic drugs, local anesthetics, beta-blockers, non-steroidal anti-inflammatory drugs and membrane-active antimicrobial agents [37,38,59]. It is useful because pharmacological effects and adverse reactions may depend not only on receptor binding but also on changes in membrane organization. In some cases, stronger perturbation of membrane order may be associated with stronger pharmacological activity or a greater risk of membrane-related toxicity.

EPR studies of membranes also support formulation research. Liposomes, micelles and lipid nanoparticles are widely used as carriers for poorly soluble or membrane-active drugs. Spin probes can reveal whether a drug or model compound is located in the aqueous phase, at the interface or within the hydrophobic domain. This information can help explain loading efficiency, release behavior and carrier stability. It also complements fluorescence and optical techniques, which may be limited in turbid or highly concentrated systems.

### 3.3. Drug Delivery Systems and Pharmaceutical Carriers

Drug delivery systems often contain domains with different polarity, viscosity and molecular mobility. These internal properties strongly influence drug loading, retention, release and stability. EPR spectroscopy is useful for studying such systems because spin probes can selectively report on the local environment inside liposomes, micelles, microemulsions, polymeric nanoparticles, hydrogels and other carriers [19,40].

A lipophilic spin probe may partition into hydrophobic regions of a carrier, whereas a hydrophilic probe remains in the aqueous phase. Changes in line shape, hyperfine splitting or rotational mobility can therefore indicate whether the probe is located in a rigid domain, a fluid lipid region or an aqueous compartment. This is useful when conventional release assays show only how much drug has been released but not what changes occur inside the carrier during dilution, swelling, erosion or storage.

**Figure 12:**
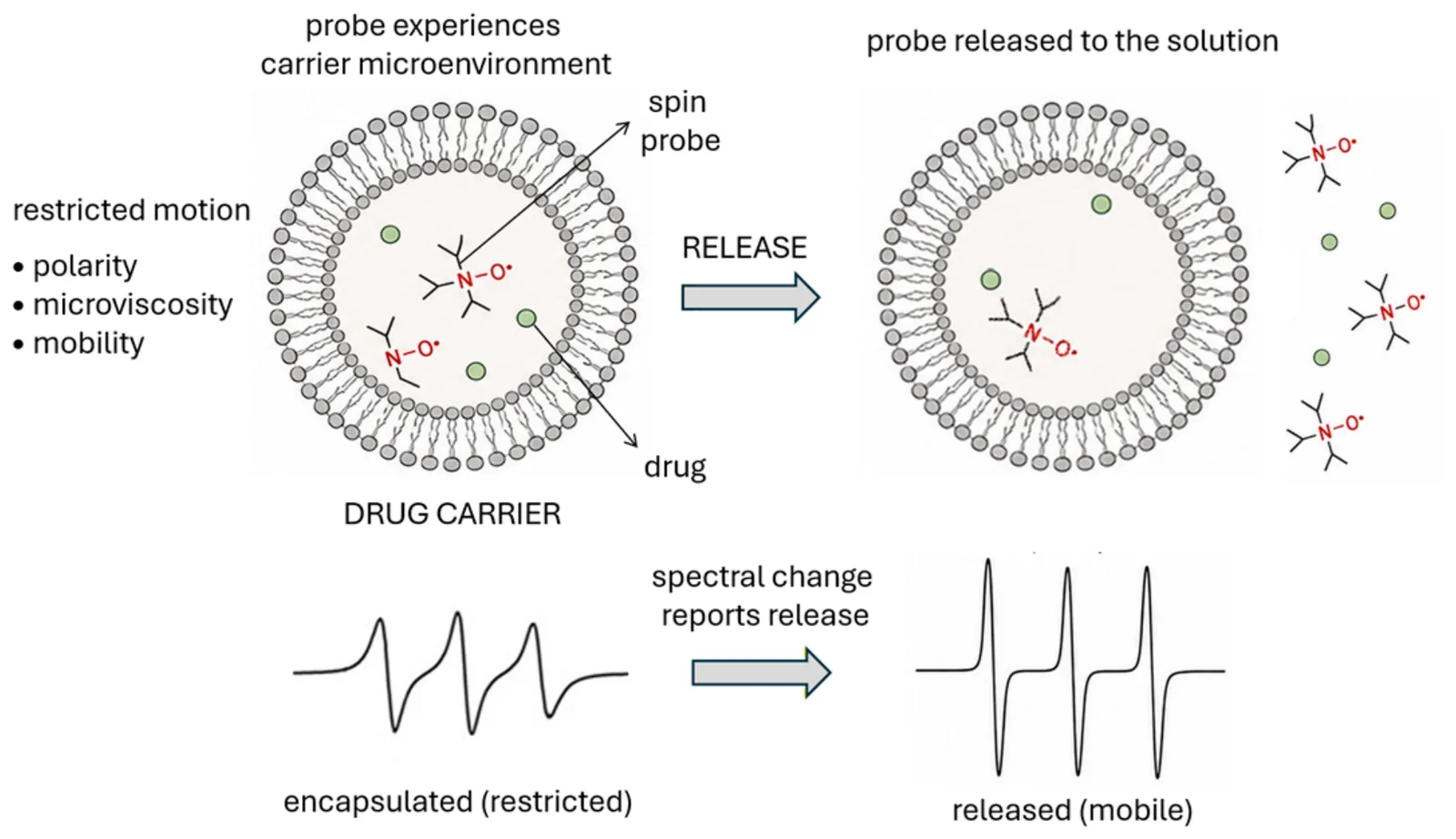
Schematic representation of drug release monitoring by EPR spectroscopy. Spectral changes of a paramagnetic probe incorporated into a delivery matrix can be used to distinguish the probe retained within the carrier from the fraction released into the surrounding medium.

EPR spectroscopy can also be used to monitor drug release mechanisms. The principle is based on incorporating a paramagnetic compound, spin-labeled model drug or nitroxide probe into a delivery matrix and following the spectral changes during release. When the probe is retained inside a solid or highly restricted matrix, its EPR spectrum reflects reduced molecular mobility. As the matrix hydrates, swells, erodes or allows diffusion of the probe into the surrounding medium, the spectral line shape changes because the released probe experiences a less restricted environment. In this way, EPR spectroscopy can distinguish between retained and released fractions of the paramagnetic compound, as schematically shown in Figure 12. This approach can provide insight into release behavior and may help distinguish between mechanisms such as erosion-controlled release from a solid delivery matrix and diffusion-controlled release from a water-containing matrix with solubilized drug [9,40].

Hydrogels provide a concrete example of this mobility-based approach. In a dry or collapsed network, an incorporated nitroxide often gives a broad, immobilized component. During water uptake, polymer plasticization and network expansion increase the narrow mobile triplet; spectral decomposition and double integration can follow the fractions of bound, confined and freely mobile probe. Subsequent chain cleavage or erosion further increases probe mobility and release, so the same measurement can distinguish early swelling from structural degradation. This strategy has been demonstrated for real-time hydrogel water-content measurements, chitosan systems containing spin-labeled insulin, bovine serum albumin gels controlling naproxen release and human serum albumin hydrogels containing a TEMPO–paullone conjugate [60,61,62,63].

Spin-labeled drug analogs and macromolecular therapeutics can also serve as direct EPR-trackable pharmacokinetic tools. A nitroxide attached to a drug, protein or carrier component can be followed through serial blood or tissue samples, or by low-frequency in vivo EPR when accessible; time-dependent signal intensity and spectral components report the distribution, tissue partitioning and apparent clearance. Examples include a nitroxide-labeled dexamethasone analog used to determine its localization in dendritic core–multishell nanoparticles and skin penetration, as well as nitroxide-labeled albumin followed in mice to resolve blood and organ pharmacokinetics [64,65]. Interpretation requires controls because the label can alter the lipophilicity, binding or transport, and biological reduction of a nitroxide to an EPR-silent hydroxylamine can mimic elimination. Comparison with the unlabeled parent and an independent concentration assay is therefore advisable.

EPR spectroscopy can also provide information on the physical stability of carrier systems during storage and after dilution in biological media. Changes in probe mobility, polarity or oxygen accessibility may indicate structural relaxation, aggregation, leakage or phase separation within the carrier. This is particularly useful for dispersed and colloidal systems, where small internal changes may occur before they become visible by particle-size measurements or conventional release testing. In this sense, EPR spectroscopy can serve as a sensitive complementary method for evaluating the structural integrity of liposomes, micelles and polymer-based carriers under formulation-relevant conditions [19,40].

Another important advantage is the ability of EPR spectroscopy to study opaque, turbid or highly concentrated samples with minimal optical interference. Many pharmaceutical carriers scatter light strongly or contain components that interfere with UV–Vis and fluorescence measurements. Because EPR spectroscopy detects the paramagnetic probe directly, it can be applied to samples that are difficult to analyze by optical techniques. This makes it particularly useful for lipid nanoparticles, concentrated emulsions, hydrogels and polymeric matrices, where local molecular mobility and microviscosity are often more informative than bulk composition alone [19,40].

EPR-based carrier studies can also support formulation optimization. By comparing spectra before and after drug loading, dilution, heating, irradiation or storage, it is possible to assess whether a drug changes the internal organization of the carrier or whether the carrier protects the drug from oxidative degradation. Such information may help in selecting excipients, optimizing carrier composition and predicting changes that affect release, stability or biological performance. Therefore, EPR spectroscopy not only describes the presence of a spin probe in a carrier but can also provide mechanistic insight into how the delivery system behaves under pharmaceutical and biologically relevant conditions [6,40].

This type of analysis is increasingly relevant for modern pharmaceutical carriers. Many delivery systems are designed to respond to pH, redox state, oxygenation, enzymatic activity or temperature. EPR-compatible probes can help evaluate whether such systems respond as intended under biologically relevant conditions. This is especially important in tumor-targeted delivery, where hypoxia, acidity and altered redox state may affect both carrier function and therapeutic response [8,9,10,11].

### 3.4. Metallodrugs and Paramagnetic Drug Systems

Metallodrugs and paramagnetic drug complexes represent another important area of application for EPR spectroscopy. Many metal-containing compounds have unpaired electrons or can form paramagnetic states during redox reactions. EPR spectroscopy can provide information on the oxidation state, coordination geometry, ligand binding, electron distribution and changes in the local environment of a metal center.

This is particularly relevant for transition-metal complexes of copper, vanadium, manganese, iron and ruthenium, as well as for lanthanide-based systems [6,12,66]. Some of these compounds are investigated as anticancer agents, insulin-mimetic compounds, redox-active drugs, imaging agents or theranostic systems [66,67,68]. EPR spectroscopy can help determine whether a metal center remains intact, undergoes ligand exchange, changes oxidation state or interacts with biological molecules [6,12,66].

A pharmaceutical connection can also be drawn from halogenated nucleobase analogs. Fluorouracil is a well-known antimetabolite used in cancer therapy, and related halogenated uracil derivatives can serve as model systems for studying metal coordination and radiation-induced paramagnetic centers [69,70]. Although 6-chlorouracil itself is primarily a model compound, its structural relationship to pharmacologically relevant uracil derivatives makes it suitable for examining how nucleobase-like molecules interact with metal ions under irradiation. In a recent single-crystal EPR study, gamma irradiation of copper-containing 6-chlorouracil crystals produced a Cu(II)–6-chlorouracil paramagnetic complex. Analysis of the complex EPR spectra, particularly the hyperfine splitting associated with the two copper isotopes and the nitrogen superhyperfine structure, enabled assignment of the local coordination environment and supported the proposed formation mechanism [71]. This example illustrates the value of EPR spectroscopy for studying radiation-induced metal complexes in molecular crystals and, more broadly, for characterizing metal centers in pharmaceutical and biologically relevant compounds.

**Figure 13:**
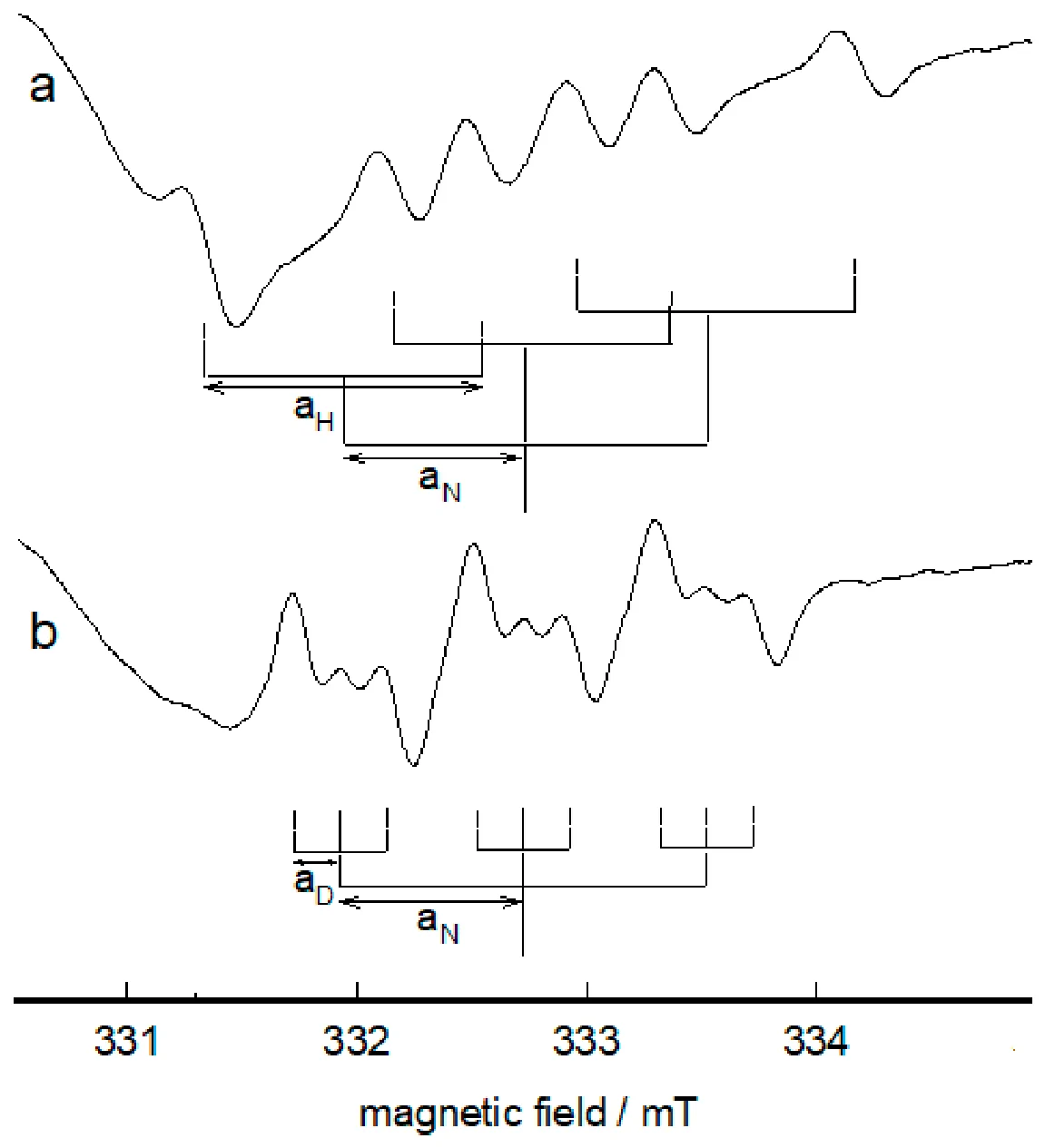
EPR spectra of the hydroxyurea-derived radical formed during reaction of hydroxyurea with vanadium(V). (**a**) Spectrum recorded in aqueous solution showing hyperfine interaction with nitrogen nucleus (*a*_N_) and additional proton coupling (*a*_H_). (**b**) Spectrum recorded in deuterated solution, where proton doublets are replaced by deuterium triplets with hyperfine constant *a*_D_.

Vanadium chemistry provides another example of the value of EPR spectroscopy in metal-mediated redox processes involving pharmacologically relevant molecules. Hydroxyurea, a clinically used antimetabolite drug in the management of myeloproliferative disorders and sickle cell disease, participates in such processes by reducing vanadium(V) to vanadium(IV) [72]. During this reaction, EPR spectroscopy enables detection of oxygen-centered hydroxyurea-derived radical intermediates, providing direct insight into the stepwise electron transfer mechanism [73]. A representative EPR spectrum of the radical formed in the vanadium(V)–hydroxyurea system is shown in Figure 13. This example highlights the pharmaceutical relevance of EPR spectroscopy for characterizing short-lived paramagnetic species generated in metal-mediated redox transformations of clinically used drugs.

Related studies with N-methylhydroxyurea and ferricyanide further show how EPR spectroscopy can follow metal-mediated electron transfer and distinguish radical pathways that depend on ligand structure and metal redox properties [74,75,76].

Beyond copper, vanadium and iron systems, manganese- and gadolinium-based complexes are also relevant to imaging and theranostic research. Their EPR interpretation, however, often requires careful consideration of the relaxation effects, oxidation state and biological matrix composition [66,72,77].

EPR spectroscopy is also valuable in the study of metalloproteins and metal-binding drugs. Metal centers often participate in electron transfer, catalysis or radical generation [12,78]. EPR spectroscopy can therefore provide mechanistic insight into both therapeutic and toxic effects. In this sense, it complements UV–Vis spectroscopy, mass spectrometry, NMR spectroscopy and X-ray-based methods.

### 3.5. EPR Oximetry and Microenvironmental Measurements

EPR oximetry is based on the sensitivity of selected paramagnetic probes to molecular oxygen. Oxygen is paramagnetic and broadens EPR spectral lines through spin exchange interactions. This oxygen-dependent line broadening provides the basis for measuring local oxygen levels in experimental systems and tissues.

This approach is particularly important in tumor biology because hypoxia influences tumor progression, radiotherapy response, immune escape and drug resistance [79,80,81]. EPR oximetry provides functional information on tissue oxygenation that complements anatomical imaging and enables direct assessment of oxygen levels in tumors [82,83,84]. In addition, EPR-based probes can be designed to report on pH, redox status, glutathione concentration and other microenvironmental parameters [8,85,86].

**Figure 14:**
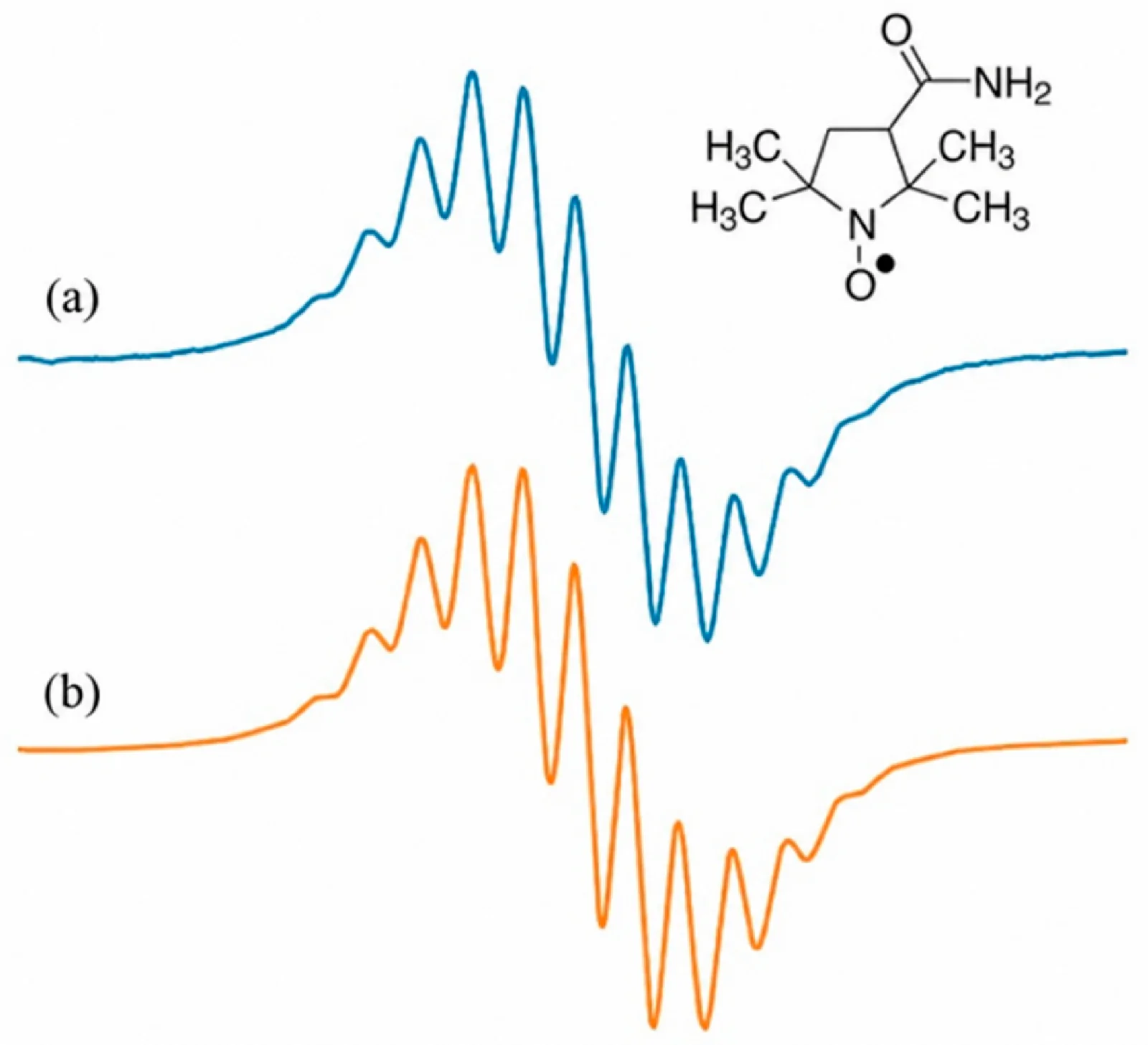
Central line of the CW-EPR spectrum of CTPO in fully deoxygenated toluene: (**a**) experimental spectrum with partially resolved superhyperfine structure (blue); (**b**) VisualEPR simulation (orange). Simulation parameters: coupling to twelve equivalent methyl protons a_H_ = 0.235 G; coupling to the C(4) proton a_H_ = 0.44 G; Lorentzian peak-to-peak linewidth ΔB_pp_ = 0.17 G.

A representative example of the well-resolved EPR spectrum of 3-carbamoyl-2,2,5,5-tetramethylpyrrolidine-1-oxyl (CTPO) under oxygen-free conditions is shown in Figure 14. In the absence of oxygen, reduced collisional broadening reveals approximately twelve partially resolved superhyperfine components in the central line. They arise principally from coupling to twelve methyl protons, with an additional contribution from the non-equivalent C(4) proton. The corresponding VisualEPR simulation reproduces this structure and illustrates how simulation supports assignment of overlapping superhyperfine contributions [87].

In oxygenated samples, molecular oxygen broadens the EPR lines. By comparing spectra recorded under deoxygenated and oxygenated conditions, this oxygen-dependent broadening can be visualized directly. In EPR oximetry, the linewidth of an oxygen-sensitive spin probe is calibrated against known oxygen concentrations or partial pressures, allowing the local oxygen level in an unknown sample to be determined from a calibration curve. Thus, the spectral linewidth serves as a quantitative indicator of the oxygen concentration in the local microenvironment.

The choice of probe is crucial for successful EPR oximetry and microenvironmental analysis. Nitroxide radicals, trityl radicals and particulate probes have been used depending on the required sensitivity, biological compatibility and time scale of measurement [82,84,88]. Nitroxides are useful because they can respond to both oxygenation and redox conditions, whereas trityl radicals provide narrow lines and high oxygen sensitivity. In pharmaceutical and biomedical research, probe selection therefore depends on whether the aim is in vitro characterization, in vivo monitoring or imaging-based assessment of tissue function [82,83,84,86].

Polarity can be read from nitroxide hyperfine splitting. A polar, protic environment and hydrogen bonding stabilize the more charge-separated resonance contribution of the N–O group and generally increase the isotropic ^14^N hyperfine coupling constant a_N_; transfer into a less polar lipid or polymer domain generally decreases a_N_. Thus, a probe calibrated in solvents of known polarity can identify whether it resides in water, at an interface or in a hydrophobic carrier compartment. If exchange between domains is slow on the EPR timescale, separate triplets can be fitted and integrated; if exchange is fast, an averaged a_N_ is observed. Probe structure, temperature and hydrogen-bond donation must be kept constant because they also influence the calibration [19,40].

Microviscosity is obtained from motion rather than bulk flow. Low-viscosity environments give narrow, nearly-equal-amplitude nitroxide lines and τ_c_ values near 10^−12^–10^−11^ s, whereas crowding, membrane ordering or polymer confinement broadens the spectrum and increases τ_c_. In the fast-motion range, outer-to-central line-height ratios and linewidths provide empirical τ_c_ estimates; anisotropic or multicomponent spectra are better analyzed by slow-motion simulation. Measuring the same probe at controlled temperature and concentration allows spectral changes to be assigned to local viscosity rather than heating or spin exchange [18,19].

pH is measured with ionizable nitroxide or trityl probes. Imidazoline and imidazolidine nitroxides contain an additional protonatable nitrogen; protonation redistributes the electron density in the nitroxide group and shifts a_N_ and, to a lesser extent, the g-value. A titration curve of a_N_ or the separation between diagnostic lines is first recorded against known pH, and an unknown local pH is then read from the calibrated response near the probe pKa. pH-sensitive trityl radicals use an analogous calibrated change in proton hyperfine splitting and offer narrower lines for in vivo measurements. These responses are probe-specific and can be affected by ionic strength, protein binding and compartmental partitioning, so matrix-matched calibration is essential [8,11,40,88].

These polarity-, viscosity- and pH-dependent observables complement oxygen-dependent linewidth broadening. For example, simultaneous EPR measurements of oxygenation and extracellular pH are valuable in acidic and hypoxic tumors, where oxygen, proton activity and redox status can change drug release, prodrug activation and treatment efficacy [8,11,84,85]. In responsive delivery systems, a calibrated probe can therefore test whether a carrier actually encounters and reacts to the intended hypoxic, acidic or viscous compartment rather than merely confirming bulk formulation composition [89,90].

EPR-based microenvironmental measurements can therefore support the design of carriers and therapeutic strategies that respond selectively to local pathological conditions rather than to average systemic conditions. EPR oximetry also has value in treatment monitoring. Tissue oxygenation may change during radiotherapy, chemotherapy, vascular-disrupting treatment or immunotherapy. Repeated EPR measurements can therefore help assess whether a treatment is modifying the tumor microenvironment in a favorable direction [91,92]. In this sense, EPR spectroscopy not only provides descriptive information but may also contribute to treatment optimization and to the development of functionally guided therapeutic approaches.

### 3.6. Current Limitations and Future Perspectives

Despite its advantages, EPR spectroscopy has several limitations. It is selective for paramagnetic species and therefore does not directly detect most diamagnetic molecules. Many pharmaceutical compounds require spin trapping, spin labeling or probe-based approaches before they become accessible to EPR analysis. In addition, spectra may be complex when several radical species, metal centers or microenvironments contribute to the signal.

Spin–spin interactions are a specific interpretive limitation. At high probe concentration or short interspin distance, dipole–dipole coupling broadens or splits lines in an orientation-dependent manner, while collision-mediated Heisenberg exchange can broaden, shift and ultimately coalesce nitroxide lines. These effects can be mistaken for increased viscosity, restricted motion, oxygen broadening or a change in polarity, and they can compromise concentration estimates if spectral integration or baseline correction is inadequate [21]. Dilution series, concentration-matched standards, low labeling densities and global simulation should therefore be used where feasible. In labeled proteins and carriers, concentration-dependent broadening should not automatically be assigned to conformational change or aggregation without these controls.

Reliable interpretation often requires appropriate controls, kinetic measurements and computer simulation. *EasySpin* is the most widely used and established software package for advanced EPR spectral simulation and analysis. At the same time, newer tools such as *VisualEPR* provide free and user-friendly environments for rapid spectral visualization and introductory simulation. In such analyses, experimental spectra are compared with simulated spectra by varying g-values, hyperfine coupling constants, linewidths, relative intensities and contributions of multiple species [23,24].

Another limitation is instrumental accessibility. Traditional EPR spectrometers require specialized infrastructure and expertise. However, recent developments in compact benchtop EPR instruments have made the technique more accessible. This is important for pharmaceutical laboratories interested in stability testing, stress studies, radical detection, antioxidant screening or quality control. Increased availability of compact instruments may therefore support the broader use of EPR spectroscopy in applied pharmaceutical research [6,57].

Emerging opportunities for EPR spectroscopy in pharmaceutical research are driven by three main developments. The first is wider access to compact instruments, which may expand the use of EPR spectroscopy beyond specialized spectroscopy laboratories and support in-line or on-site monitoring in pharmaceutical development and manufacturing. The second is the development of more selective spin probes, spin labels and oxygen- or pH-sensitive paramagnetic reporters, which may improve the analysis of drug delivery systems, biological microenvironments and treatment response. The third is the growing use of spectral simulation, digital tools and automated data analysis, which can make the interpretation of complex EPR spectra more reproducible and accessible to non-specialist users.

Together, these developments may strengthen the role of EPR spectroscopy as a complementary method in pharmaceutical development. However, EPR spectroscopy is unlikely to become a universal routine method for all pharmaceutical analyses. Its main value lies in targeted questions where radical chemistry, oxidative degradation, paramagnetic metal centers, oxygenation, local microviscosity or other microenvironmental properties are central to the problem being investigated.

## 4. Conclusions

Electron paramagnetic resonance (EPR) spectroscopy is a specialized but highly informative analytical method in pharmaceutical research. Its main strength is the direct detection and characterization of paramagnetic species, including free radicals, transition-metal centers, spin labels and spin probes. For this reason, EPR spectroscopy is particularly valuable in studies where radical chemistry, redox processes, oxidative degradation, molecular mobility or local microenvironmental properties are central to the research question.

The reviewed applications show that EPR spectroscopy can support several areas of pharmaceutical science. Spin trapping enables the detection of short-lived radical intermediates involved in drug action, toxicity, photochemical reactions and synthetic transformations relevant to medicinal chemistry. EPR-based antioxidant assays provide direct information on radical-scavenging activity, including complex samples that may be difficult to analyze by optical methods. Spin labels and spin probes allow the investigation of membranes, pharmaceutical carriers, microviscosity, polarity, oxygenation and drug release mechanisms. In addition, EPR spectroscopy contributes to the characterization of metallodrugs, paramagnetic complexes and tissue microenvironments, including oxygen, pH and redox status.

EPR spectroscopy should not be regarded as a universal routine method for all pharmaceutical analyses. Its value is greatest when conventional analytical methods cannot directly answer questions involving unpaired electrons, radical intermediates, paramagnetic metal centers or local molecular dynamics. Recent progress in compact CW-EPR instruments, spectral simulation tools, selective spin probes and microenvironment-sensitive reporters may further expand its use in pharmaceutical development, stability testing, drug delivery research and treatment monitoring.

Overall, EPR spectroscopy provides a methodological bridge between radical chemistry, pharmaceutical technology, toxicology, biophysics and biomedical research. By linking molecular-level paramagnetic processes with drug efficacy, safety, stability and delivery, EPR spectroscopy has strong potential to remain an important complementary tool in current and emerging areas of pharmaceutical research.

## Data Availability

No new data were created or analyzed in this study. Data sharing is not applicable.

## Abbreviations

The following abbreviations are used in this manuscript:

5-, 12-, 16-DS	5-, 12-, 16-doxyl stearic acid
BMPO	5-tert-butoxycarbonyl-5-methyl-1-pyrroline N-oxide
CTPO	3-carbamoyl-2,2,5,5-tetramethylpyrrolidine-1-oxyl
CW-EPR	continuous-wave electron paramagnetic resonance
DEPMPO	5-(diethoxyphosphoryl)-5-methyl-1-pyrroline N-oxide
DMPO	5,5-dimethyl-1-pyrroline N-oxide
DMPO-OH	DMPO-hydroxyl radical adduct
DMPO-OOH	DMPO-superoxide radical adduct
DNA	deoxyribonucleic acid
DPPH	2,2-diphenyl-1-picrylhydrazyl
EPR	electron paramagnetic resonance
NMR	nuclear magnetic resonance
PBN	N-tert-butyl-α-phenylnitrone
ROS	reactive oxygen species
TEMPO	2,2,6,6-tetramethylpiperidine-1-oxyl
TEMPOL	4-hydroxy-2,2,6,6-tetramethylpiperidine-1-oxyl
UV	ultraviolet
UV–Vis	ultraviolet–visible spectroscopy

## References

[1] Sies H., Sies H. (2020). Oxidative eustress and oxidative distress: Introductory remarks. Oxidative Stress: Eustress and Distress.

[2] Juan C.A., Pérez de la Lastra J.M., Plou F.J., Pérez-Lebeña E. (2021). The chemistry of reactive oxygen species (ROS) revisited: Outlining their role in biological macromolecules (DNA, lipids and proteins) and induced pathologies. Int. J. Mol. Sci..

[3] Jomova K., Raptova R., Alomar S.Y., Alwasel S.H., Nepovimova E., Kuca K., Valko M. (2023). Reactive oxygen species, toxicity, oxidative stress, and antioxidants: Chronic diseases and aging. Arch. Toxicol..

[4] Iravani S., Soufi G.J. (2020). Electron paramagnetic resonance (EPR) spectroscopy: Food, biomedical and pharmaceutical analysis. Biomed. Spectrosc. Imaging.

[5] Davies M.J. (2016). Detection and characterisation of radicals using electron paramagnetic resonance (EPR) spin trapping and related methods. Methods.

[6] Bešić E., Rajić Z., Šakić D. (2024). Advancements in electron paramagnetic resonance (EPR) spectroscopy: A comprehensive tool for pharmaceutical research. Acta Pharm..

[7] Schöneich C. (2023). Primary processes of free radical formation in pharmaceutical formulations of therapeutic proteins. Biomolecules.

[8] Bobko A.A., Eubank T.D., Driesschaert B., Khramtsov V.V. (2018). In vivo EPR assessment of pH, pO_2_, redox status, and concentrations of phosphate and glutathione in the tumor microenvironment. J. Vis. Exp..

[9] Chumakova N.A., Golubeva E.N., Kuzin S.V., Ivanova T.A., Grigoriev I.A., Kostjuk S.V., Melnikov M.Y. (2020). New insight into the mechanism of drug release from poly(lactic-co-glycolic acid) microparticles using electron paramagnetic resonance spectroscopy. Polymers.

[10] Rajendran A., Philip S., Elumalai V., Srinivasan S., Elumalai K. (2026). Molecular imaging biomarkers of the tumor microenvironment: Advances in early cancer detection and treatment monitoring. Adv. Biomark. Sci. Technol..

[11] Buyse C., Mignion L., Joudiou N., Melloul S., Driesschaert B., Gallez B. (2024). Sensitive simultaneous measurements of oxygenation and extracellular pH by EPR using a stable monophosphonated trityl radical and lithium phthalocyanine. Free Radic. Biol. Med..

[12] Augusto O., Truzzi D.R., Linares E. (2023). Electron paramagnetic resonance (EPR) for investigating relevant players of redox reactions: Radicals, metalloproteins and transition metal ions. Redox Biochem. Chem..

[13] Spasojević I. (2011). Free radicals and antioxidants at a glance using EPR spectroscopy. Crit. Rev. Clin. Lab. Sci..

[14] Hawkins C.L., Davies M.J. (2014). Detection and characterisation of radicals in biological materials using EPR methodology. Biochim. Biophys. Acta.

[15] Gordy W. (1980). Theory and Applications of Electron Spin Resonance.

[16] Zubčić G., You J., Zott F.L., Ashirbaev S.S., Kolympadi Marković M., Bešić E., Vrček V., Zipse H., Šakić D. (2024). Regioselective rearrangement of nitrogen- and carbon-centered radical intermediates in the Hofmann–Löffler–Freytag reaction. J. Phys. Chem. A.

[17] Zubčić G., Andrijanić L., Džeba I., You J., Friganović T., Portada T., Pavić K., Bešić E., Vrček V., Šakić D. (2025). Mechanistic insights into the propagation cycle of the Hofmann–Löffler–Freytag reaction: Halogen vs hydrogen atom transfer. J. Org. Chem..

[18] Zubčić G., Pavić K., You J., Vrček V., Portada T., Bešić E., Šakić D. (2025). Light-induced rearrangement from macrocyclic to bicyclic lactam: A case study of N-chlorinated laurolactam. Acta Pharm..

[19] Uddin M.A., Yu H., Wang L., Naveed K., Haq F., Amin B., Mehmood S., Nazir A., Xing Y., Shen D. (2020). Recent progress in EPR study of spin labeled polymers and spin probed polymer systems. J. Polym. Sci..

[20] Torricella F., Pierro A., Mileo E., Belle V., Bonucci A. (2021). Nitroxide spin labels and EPR spectroscopy: A powerful association for protein dynamics studies. Biochim. Biophys. Acta Proteins Proteom..

[21] Bales B.L., Meyer M., Smith S., Peric M. (2009). EPR line shifts and line shape changes due to spin exchange and dipole–dipole interactions of nitroxide free radicals in liquids: 5. Further experimental and theoretical efforts to separate the effects of the two interactions. J. Phys. Chem. A.

[22] Smirnov A.I., Belford R.L. (1995). Rapid quantitation from inhomogeneously broadened EPR spectra by a fast convolution algorithm. J. Magn. Reson. A.

[23] Stoll S., Schweiger A. (2006). EasySpin, a comprehensive software package for spectral simulation and analysis in EPR. J. Magn. Reson..

[24] Šakić D., Bešić E., Zubčić G. VisualEPR, Online EPR Spectra Visualization and Simulation Tool. https://github.com/DSakicLab/visualEPR.

[25] Abbas K., Babić N., Peyrot F. (2016). Use of spin traps to detect superoxide production in living cells by electron paramagnetic resonance (EPR) spectroscopy. Methods.

[26] Villamena F.A., Zweier J.L. (2004). Detection of reactive oxygen and nitrogen species by EPR spin trapping. Antioxid. Redox Signal..

[27] Fontmorin J.M., Burgos Castillo R.C., Sillanpää M. (2016). Stability of 5,5-dimethyl-1-pyrroline-N-oxide as a spin-trap for quantification of hydroxyl radicals in processes based on Fenton reaction. Water Res..

[28] Braxton E.M., Fox D.J., Breez B.G., Tully J.J., Levey K.J., Newton M.E., Macpherson J.V. (2022). Electron paramagnetic resonance for the detection of electrochemically generated hydroxyl radicals: Issues associated with electrochemical oxidation of the spin trap. ACS Meas. Sci. Au.

[29] Wang L., Cao J., Wang P., Fu Y., Chen J., Wang Z. (2024). Hydroperoxide-independent generation of spin trapping artifacts by quinones and DMPO: Implication for radical identification in quinone-related reactions. Environ. Health.

[30] Roubaud V., Sankarapandi S., Kuppusamy P., Tordo P., Zweier J.L. (1997). Quantitative measurement of superoxide generation using the spin trap 5-(diethoxyphosphoryl)-5-methyl-1-pyrroline-N-oxide. Anal. Biochem..

[31] Mittag J.J., Trutschel M.-L., Kruschwitz H., Mäder K., Buske J., Garidel P. (2022). Characterization of radicals in polysorbate 80 using electron paramagnetic resonance (EPR) spectroscopy and spin trapping. Int. J. Pharm. X.

[32] Rahmani-Manglano N.E., Fallahasghari E.Z., Mendes A.C., Andersen M.L., Guadix E.M., Chronakis I.S., García-Moreno P.J. (2024). Oxidative stability of fish oil-loaded nanocapsules produced by electrospraying using kafirin or zein proteins as wall materials. Antioxidants.

[33] Di Prima G., De Caro V., Cardamone C., Oliveri G., D’Oca M.C. (2024). EPR spectroscopy coupled with spin trapping as an alternative tool to assess and compare the oxidative stability of vegetable oils for cosmetics. Appl. Sci..

[34] Victória H.F.V., Ferreira D.C., Filho J.B.G., Martins D.C.S., Pinheiro M.V.B., Sáfar G.A.M., Krambrock K. (2022). Detection of singlet oxygen by EPR: The instability of the nitroxyl radicals. Free Radic. Biol. Med..

[35] Moghadam M.S., Wiens E., Gauvrit S., Sammynaiken R., Collins M.M. (2025). Electron paramagnetic resonance spectroscopy for analysis of free radicals in zebrafish. PLoS ONE.

[36] Berliner L.J., Reuben J. (1989). Spin Labeling: Theory and Applications.

[37] Sahu I.D., Lorigan G.A. (2020). Electron paramagnetic resonance as a tool for studying membrane proteins. Biomolecules.

[38] Sahu I.D., Lorigan G.A. (2021). Probing structural dynamics of membrane proteins using electron paramagnetic resonance spectroscopic techniques. Biophysica.

[39] Ledwitch K.V., Künze G., Okwei E., Sala D., Meiler J. (2024). Non-canonical amino acids for site-directed spin labeling of membrane proteins. Curr. Opin. Struct. Biol..

[40] Gallez B. (2026). Electron paramagnetic resonance (EPR) meets drug delivery and biomaterials: A magnetic love story. Drug Deliv. Transl. Res..

[41] Sies H., Berndt C., Jones D.P. (2017). Oxidative Stress. Annu. Rev. Biochem..

[42] Williams H.E., Loades V.C., Claybourn M., Murphy D.M. (2006). Electron paramagnetic resonance spectroscopy studies of oxidative degradation of an active pharmaceutical ingredient and quantitative analysis of the organic radical intermediates using partial least-squares regression. Anal. Chem..

[43] Schieber M., Chandel N.S. (2014). ROS function in redox signaling and oxidative stress. Curr. Biol..

[44] Polak J., Bartoszek M., Stanimirova I. (2013). A study of the antioxidant properties of beers using electron paramagnetic resonance. Food Chem..

[45] Romanet R., Sarhane Z., Bahut F., Uhl J., Schmitt-Kopplin P., Nikolantonaki M., Gougeon R.D. (2021). Exploring the chemical space of white wine antioxidant capacity: A combined DPPH, EPR and FT-ICR-MS study. Food Chem..

[46] Zalibera M., Staško A., Šlebodová A., Jančovičová V., Čermáková T., Brezová V. (2008). Antioxidant and radical-scavenging activities of Slovak honeys—An electron paramagnetic resonance study. Food Chem..

[47] Shakoori Z., Salaseh E., Mehrabian A.R., Tehrani D.M., Dardashti N.F., Salmanpour F. (2024). The amount of antioxidants in honey has a strong relationship with the plants selected by honey bees. Sci. Rep..

[48] Merkx D.W.H., Plankensteiner L., Yu Y., Wierenga P.A., Hennebelle M., van Duynhoven J.P.M. (2021). Evaluation of PBN spin-trapped radicals as early markers of lipid oxidation in mayonnaise. Food Chem..

[49] Kozłowska M., Zawada K. (2015). Evaluation of oxidative stability of vegetable oils enriched with herb extracts by EPR spectroscopy. Chem. Pap..

[50] Alberti A., Bolognese A., Guerra M., Lavecchia A., Macciantelli D., Marcaccio M., Novellino E., Paolucci F. (2003). Antitumor agents 4. Characterization of free radicals produced during reduction of the antitumor drug 5H-pyridophenoxazin-5-one: An EPR study. Biochemistry.

[51] Butler A.R., Gilbert B.C., Hulme P., Irvine L.R., Renton L., Whitwood A.C. (1998). EPR evidence for the involvement of free radicals in the iron-catalysed decomposition of qinghaosu (artemisinin) and some derivatives; antimalarial action of some polycyclic endoperoxides. Free Radic. Res..

[52] Asano M., Iwahashi H. (2017). Determination of the structures of radicals formed in the reaction of antimalarial drug artemisinin with ferrous ions. Eur. J. Med. Chem..

[53] Poje G., Šakić D., Marinović M., You J., Tarpley M., Williams K.P., Golub N., Dernovšek J., Tomašič T., Bešić E. (2024). Unveiling the antiglioblastoma potential of harmicens, harmine and ferrocene hybrids. Acta Pharm..

[54] Suzen S., Gurer-Orhan H., Saso L. (2017). Detection of reactive oxygen and nitrogen species by electron paramagnetic resonance (EPR) technique. Molecules.

[55] de Menezes S.L., Augusto O. (2001). EPR detection of glutathionyl and protein-tyrosyl radicals during the interaction of peroxynitrite with macrophages. J. Biol. Chem..

[56] Bruker Seeing the Unseen: Degradation Measured by EPR Spectroscopy. Application Note. https://www.syntechinnovation.com/images/Knowledge/mat_epr/Degradation_measured_by_EPR_spectroscopy.pdf.

[57] Bruker Pharmaceutical Applications of EPR Spectroscopy. Webinar, 2018. https://www.bruker.com/de/news-and-events/webinars/2018/pharmaceutical-applications-of-epr-spectroscopy.html.

[58] Subczynski W.K., Widomska J., Feix J.B. (2009). Physical properties of lipid bilayers from EPR spin labeling and their influence on chemical reactions in a membrane environment. Free Radic. Biol. Med..

[59] de Paula E., Schreier S. (1995). Use of a novel method for determination of partition coefficients to compare the effect of local anesthetics on membrane structure. Biochim. Biophys. Acta.

[60] Vesković A., Nakarada Đ., Popović Bijelić A. (2021). A novel methodology for hydrogel water content determination by EPR: The basis for real-time monitoring of controlled drug release and hydrogel swelling and degradation. Polym. Test..

[61] Kempe S., Metz H., Bastrop M., Hvilsom A., Contri R.V., Mäder K. (2008). Characterization of thermosensitive chitosan-based hydrogels by rheology and electron paramagnetic resonance spectroscopy. Eur. J. Pharm. Biopharm..

[62] Sanaeifar N., Mäder K., Hinderberger D. (2022). Macro- and nanoscale effect of ethanol on bovine serum albumin gelation and naproxen release. Int. J. Mol. Sci..

[63] Vesković A., Nakarada Đ., Vasiljević O., Dobrov A., Spengler G., Enyedy É.A., Arion V.B., Popović Bijelić A. (2022). The release of a highly cytotoxic paullone bearing a TEMPO free radical from the HSA hydrogel: An EPR spectroscopic characterization. Pharmaceutics.

[64] Saeidpour S., Lohan S.B., Anske M., Unbehauen M., Fleige E., Haag R., Meinke M.C., Bittl R., Teutloff C. (2017). Localization of dexamethasone within dendritic core–multishell (CMS) nanoparticles and skin penetration properties studied by multi-frequency electron paramagnetic resonance (EPR) spectroscopy. Eur. J. Pharm. Biopharm..

[65] Liebmann J., Bourg J., Krishna C.M., Glass J., Cook J.A., Mitchell J.B. (1994). Pharmacokinetic properties of nitroxide-labeled albumin in mice. Life Sci..

[66] Smith T.S., LoBrutto R., Pecoraro V.L. (2002). Paramagnetic spectroscopy of vanadyl complexes and its applications to biological systems. Coord. Chem. Rev..

[67] Alessio E., Messori L. (2019). NAMI-A and KP1019/1339, two iconic ruthenium anticancer drug candidates face-to-face: A case story in medicinal inorganic chemistry. Molecules.

[68] Sharfalddin A.A., Al-Younis I.M., Mohammed H.A., Dhahri M., Mouffouk F., Abu Ali H., Anwar M.J., Qureshi K.A., Hussien M.A., Alghrably M. (2022). Therapeutic properties of vanadium complexes. Inorganics.

[69] Sara J.D., Kaur J., Khodadadi R., Rehman M., Lobo R., Chakrabarti S., Herrmann J., Lerman A., Grothey A. (2018). 5-fluorouracil and cardiotoxicity: A review. Ther. Adv. Med. Oncol..

[70] Denifl S., Ptasińska S., Gstir B., Scheier P., Märk T.D. (2004). Electron impact ionization of 5- and 6-chlorouracil: Appearance energies. Int. J. Mass Spectrom..

[71] Šakić D., Bešić E. (2025). EPR study of a copper impurity center in a single crystal of 6-chlorouracil. J. Mol. Struct..

[72] Lagostina V., Regueiro-Figueroa M., Rousselin Y., Esteban-Gómez D., Platas-Iglesias C., Tripier R. (2023). Magnetic and relaxation properties of vanadium(IV) complexes: An integrated ^1^H relaxometric, EPR and computational study. Inorg. Chem. Front..

[73] Gabričević M., Bešić E., Biruš M., Zahl A., van Eldik R. (2006). Oxidation of hydroxyurea with oxovanadium(V) ions in acidic aqueous solution. J. Inorg. Biochem..

[74] Weitner T., Bešić E., Kos I., Gabričević M., Biruš M. (2007). Formation of free radicals during the oxidation of N-methylhydroxyurea with dioxovanadium(V) ion. Tetrahedron Lett..

[75] Budimir A., Bešić E., Biruš M. (2009). Kinetics and mechanism of oxidation of hydroxyurea with hexacyanoferrate(III) ions in aqueous solution. Croat. Chem. Acta.

[76] Budimir A., Weitner T., Kos I., Šakić D., Gabričević M., Bešić E., Biruš M. (2011). Kinetics and mechanism of oxidation of hydroxyurea derivatives with hexacyanoferrate(III) in aqueous solution. Croat. Chem. Acta.

[77] Danhier P., Gallez B. (2014). Electron paramagnetic resonance: A powerful tool to support magnetic resonance imaging research. Contrast Media Mol. Imaging.

[78] Jasniewski A., Hu Y., Ribbe M.W. (2019). Electron paramagnetic resonance spectroscopy of metalloproteins. Methods Mol. Biol..

[79] Boulefour W., Rowinski E., Louati S., Sotton S., Wozny A.S., Moreno-Acosta P., Mery B., Rodriguez-Lafrasse C., Magne N. (2021). A review of the role of hypoxia in radioresistance in cancer therapy. Med. Sci. Monit..

[80] Mortezaee K., Majidpoor J. (2021). The impact of hypoxia on immune state in cancer. Life Sci..

[81] Bigos K.J., Quiles C.G., Lunj S., Smith D.J., Krause M., Troost E.G., West C.M., Hoskin P., Choudhury A. (2024). Tumour response to hypoxia: Understanding the hypoxic tumour microenvironment to improve treatment outcome in solid tomours. Front. Oncol..

[82] Epel B., Kotecha M., Halpern H.J. (2017). In vivo preclinical cancer and tissue engineering applications of absolute oxygen imaging using pulse EPR. J. Magn. Reson..

[83] Matsumoto K., Mitchell J.B., Krishna M.C. (2021). Multimodal functional imaging for cancer/tumor microenvironments based on MRI, EPR, and PET. Molecules.

[84] Schaner P.E., Williams B.B., Chen E.Y., Pettus J.R., Schreiber W.A., Kmiec M.M., Jarvis L.A., Pastel D.A., Zuurbier R.A., DiFlorio-Alexander R.M. (2021). First-in-human study in cancer patients establishing the feasibility of oxygen measurements in tumors using electron paramagnetic resonance with the OxyChip. Front. Oncol..

[85] Roshchupkina G.I., Bobko A.A., Bratasz A., Reznikov V.A., Kuppusamy P., Khramtsov V.V. (2008). In vivo EPR measurement of glutathione in tumor-bearing mice using improved disulfide biradical probe. Free Radic. Biol. Med..

[86] Babić N., Peyrot F. (2019). Molecular probes for evaluation of oxidative stress by in vivo EPR spectroscopy and imaging: State-of-the-art and limitations. Magnetochemistry.

[87] Hyde J.S., Subczynski W.K. (1984). Simulation of ESR spectra of oxygen-sensitive spin-label probe CTPO. J. Magn. Reson..

[88] Voinov M.A., Smirnova T.I., Smirnov A.I. (2021). EPR oximetry with nitroxides: Effects of molecular structure, pH, and electrolyte concentration. Appl. Magn. Reson..

[89] Li Y., Jeon J., Park J.H. (2020). Hypoxia-responsive nanoparticles for tumor-targeted drug delivery. Cancer Lett..

[90] Saadh M.J., Mustafa M.A., Qassem L.Y., Ghadir G.K., Alaraj M., Alubiady M.H.S., Al-Abdeen S.H.Z., Shakier H.G., Alshahrani M.Y., Zwamel A.H. (2024). Targeting hypoxic and acidic tumor microenvironment by nanoparticles: A review. J. Drug Deliv. Sci. Techn..

[91] Šentjurc M., Čemažar M., Serša G. (2004). EPR oximetry of tumors in vivo in cancer therapy. Spectrochim. Acta A Mol. Biomol. Spectrosc..

[92] Hou H., Lariviere J.P., Deminenko E., Gladstone D., Swartz H., Khan N. (2009). Repeated tumor pO_2_ measurements by multi-site EPR oximetry as a prognostic marker for enhanced therapeutic efficacy of fractionated radiotherapy. Radiother. Oncol..

